# Acid‐Resistant BODIPY Amino Acids for Peptide‐Based Fluorescence Imaging of GPR54 Receptors in Pancreatic Islets

**DOI:** 10.1002/anie.202302688

**Published:** 2023-04-12

**Authors:** Lorena Mendive‐Tapia, Laia Miret‐Casals, Nicole D. Barth, Jinling Wang, Anne de Bray, Massimiliano Beltramo, Vincent Robert, Christophe Ampe, David J. Hodson, Annemieke Madder, Marc Vendrell

**Affiliations:** ^1^ Centre for Inflammation Research The University of Edinburgh EH16 4TJ Edinburgh UK; ^2^ Department of Organic and Macromolecular Chemistry Faculty of Sciences Ghent University 9000 Ghent Belgium; ^3^ Oxford Centre for Diabetes Endocrinology and Metabolism (OCDEM) Radcliffe Department of Medicine University of Oxford OX3 7LE Oxford UK; ^4^ Equipe Neuroendocrinologie Moleculaire de la Reproduction Physiologie de la Reproduction et des Comportements Centre INRA Val de Loire 37380 Nouzilly France; ^5^ Department of Biomolecular Medicine Faculty of Medicine and Health Sciences Ghent University 9052 Ghent Belgium

**Keywords:** Diabetes, Fluorescence, GPCRs, Probes, Solid-Phase Peptide Synthesis

## Abstract

The G protein‐coupled kisspeptin receptor (GPR54 or KISS1R) is an important mediator in reproduction, metabolism and cancer biology; however, there are limited fluorescent probes or antibodies for direct imaging of these receptors in cells and intact tissues, which can help to interrogate their multiple biological roles. Herein, we describe the rational design and characterization of a new acid‐resistant BODIPY‐based amino acid (Trp‐BODIPY PLUS), and its implementation for solid‐phase synthesis of fluorescent bioactive peptides. Trp‐BODIPY PLUS retains the binding capabilities of both short linear and cyclic peptides and displays notable turn‐on fluorescence emission upon target binding for wash‐free imaging. Finally, we employed Trp‐BODIPY PLUS to prepare some of the first fluorogenic kisspeptin‐based probes and visualized the expression and localization of GPR54 receptors in human cells and in whole mouse pancreatic islets by fluorescence imaging.

## Graphical abstract

We describe Trp‐BODIPY PLUS as a new acid‐resistant building block for straightforward solid‐phase synthesis of fluorogenic peptides. We demonstrate the utility of Trp‐BODIPY PLUS with the first fluorogenic probe for direct visualization and quantitative analysis of the expression and localization of the G protein‐coupled receptors 54 (GPR54 or kisspeptin) in human cells and intact mouse pancreatic islets.

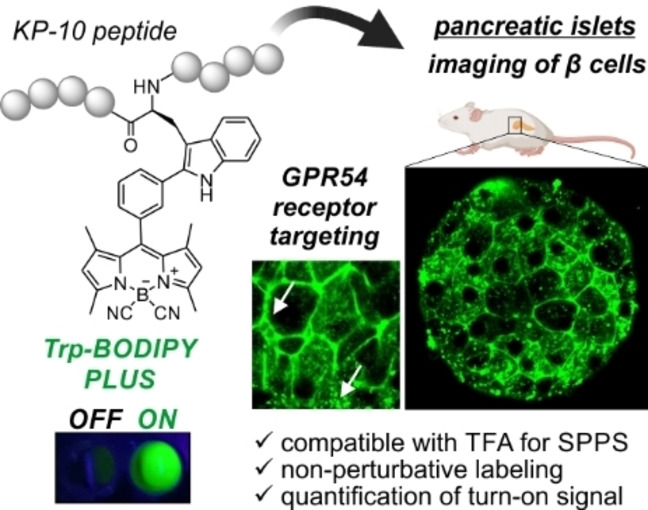

## Introduction

G protein‐coupled receptors (GPCRs) are the largest family of membrane proteins involved in cellular signaling pathways.^[1]^ In particular, GPR54 (also known as KISS1R) and their cognate peptide ligands kisspeptins play essential roles in the regulation of puberty and reproductive function, metabolism and cancer biology.^[2]^ Thus, loss‐of‐function mutations in GPR54 are associated with delayed puberty and infertility due to hypogonadotropic hypogonadism.^[3]^ Moreover, GPR54 complexes mediate anti‐metastatic responses in different cancers and its downregulation is associated with poor prognosis.^[4]^ Kisspeptin signaling is also a key regulator of insulin secretion in pancreatic islets,^[5]^ suggesting that the expression and localization of GPR54 receptors can be used as β cell biomarkers.

Classical approaches for the detection of GPR54 mostly rely on RT‐PCR gene expression, Cre‐reporter approaches, immunohistochemical methods using transfected cells or radiolabeled ligands.^[6]^ Fluorescent peptides are powerful and low‐cost tools to interrogate biological processes at a cellular level with high spatiotemporal resolution.^[7]^ In this regard, only a few number of fluorescent probes for direct imaging of GPR54 have been reported to date,^[8]^ which highlights the need of new tools that can provide further insights into their physiological behavior in live cells and tissues (e.g., pancreatic islets).

**Scheme 1:**
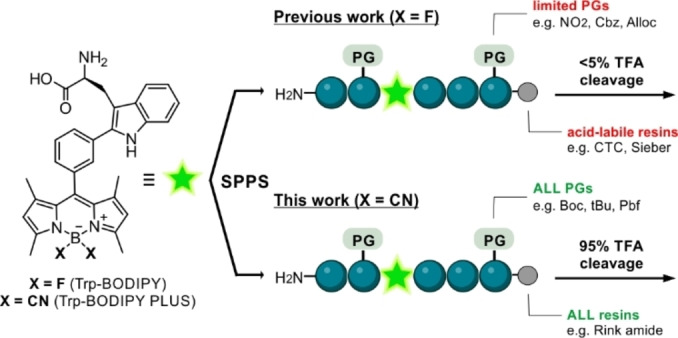
Chemical strategies for the synthesis of BODIPY‐based fluorogenic amino acids and solid‐phase peptide synthesis (SPPS) of fluorescent conjugates.

The development of non‐natural fluorogenic amino acids (FlAAs) as building blocks for the modification of peptides and proteins has opened new avenues for wash‐free fluorescence imaging.^[9]^ Our group has described several FlAAs with distinct chemical and optical features (e.g., small size, super‐resolution capability, fluorogenicity) for live‐cell imaging.^[10]^ Some of these FlAAs are based on boron‐dipyrromethene derivatives of tryptophan (Trp‐BODIPY) (Scheme 1), which display notable environmental sensitivity and good signal‐to‐noise ratios. However, the lability of the BODIPY core to acid media (e.g., trifluoroacetic acid, TFA) limits its implementation in solid‐phase peptide synthesis (SPPS)^[11]^ for the fast generation of fluorescent peptides. In the present work, we have overcome this limitation with the rational development of Trp‐BODIPY PLUS (Scheme 1) as the first TFA‐resistant BODIPY‐based FlAA for conventional SPPS, while maintaining its compact size and fluorogenicity. We systematically demonstrated the remarkable stability of Trp‐BODIPY PLUS under standard conditions used in SPPS, and its application to synthesize environmentally sensitive peptides that retain the molecular recognition features of their native analogues. Furthermore, we employed this technology to synthesize some of the first fluorogenic probes for selective imaging of GPR54 in human cells and mouse pancreatic islets. To the best of our knowledge, this is the first example of peptide based fluorescent probes for targeted imaging of GPR54‐expressing cell subpopulations in intact pancreatic islets.

## Results and Discussion

### Design, synthesis and characterization of acid‐resistant BODIPY fluorogenic amino acids

The most conventional strategies for labeling peptides with BODIPY fluorophores^[12]^ make use of couplings between reactive dyes (e.g., NHS esters) and N‐terminal groups or amine‐functionalized side chains (e.g., lysine).^[13]^ In order to facilitate the introduction of BODIPY dyes at non‐polar residues, our group reported the direct incorporation of the Trp‐BODIPY FlAAs into peptide sequences using SPPS. The advantages of this approach are two‐fold: 1) to enable site‐selective modification of peptides without altering polar groups or bioactivity,[^10e^, ^10f^, ^10g^, ^10h^] 2) to introduce a turn‐on reporter within the peptide sequence able to directly monitor target binding. However, the poor stability of Trp‐BODIPY in TFA solutions requires synthetic procedures that employ non‐acid labile protecting groups (PG) and acid‐sensitive resins (Scheme 1). To overcome this limitation and expand the general applicability of BODIPY fluorophores to most PGs and solid supports for SPPS, we studied the derivatization of BODIPY FlAAs with groups that could increase their resistance to TFA.

We decided to modify the acid‐labile boron center of the BODIPY core by replacing the two fluorine atoms with groups that would reduce the lability of the B−N bonds in acid media.^[14]^ Considering the chemical stability and accessibility of trimethylsilyl (TMS)‐nucleophiles, we subjected Trp‐BODIPY to a screening with commercial nucleophiles in the presence of BF_3_⋅OEt_2_ as the Lewis acid (Table 1). Attempts to generate B−O and B−N bonds with TMS‐OAc, TMS‐N(CH_3_)_2_ and TMS‐N_3_ only led to traces of decomposed amino acid (entries A–C, Table 1). Treatment with TMS‐CF_3_ and TMS‐C≡CH to form B−C bonds resulted in higher rates of BODIPY decomposition with no substantial formation of the final products (entries D–E, Table 1). Finally, the reaction between Trp‐BODIPY and TMS‐CN afforded the substitution product with ≈70 % conversion (entry F, Table 1). Interestingly, reducing the reaction time from 1 h to only 10 min led to >90 % conversion with minimal BODIPY decomposition (entry G, Table 1). This behavior may be attributed to the equilibrium between the starting fluorinated precursor and the substitution product due to the excess of BF_3_⋅OEt_2_. Further attempts to react Trp‐BODIPY and TMS‐protected nucleophiles with other Lewis acids (e.g., SnCl_4_, BCl_3_)^[15]^ were not successful (Table S1); therefore, we scaled up and isolated the cyano‐derivative Trp‐BODIPY PLUS in good yields (58 %) and >95 % purity for further characterization (full details in Supporting Information).

**Table 1:**
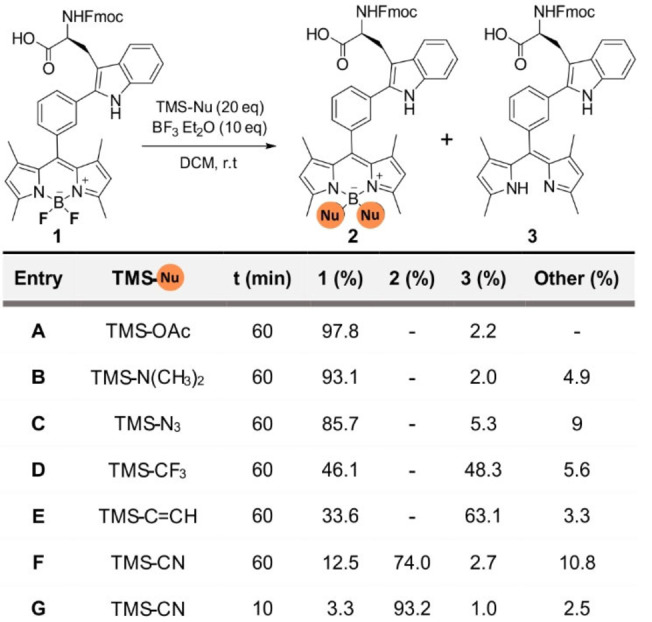
Screening of TMS‐protected nucleophiles (TMS‐Nu) with the Trp‐BODIPY scaffold to expand diversification at the boron centre.^[a,b]^

[a] Reaction conditions: Trp‐BODIPY (5 mM), BF_3_⋅Et_2_O (10 equiv), TMS‐nucleophile (20 equiv), DCM, r.t. [b] Conversion determined by HPLC‐MS [%].

**Figure 1:**
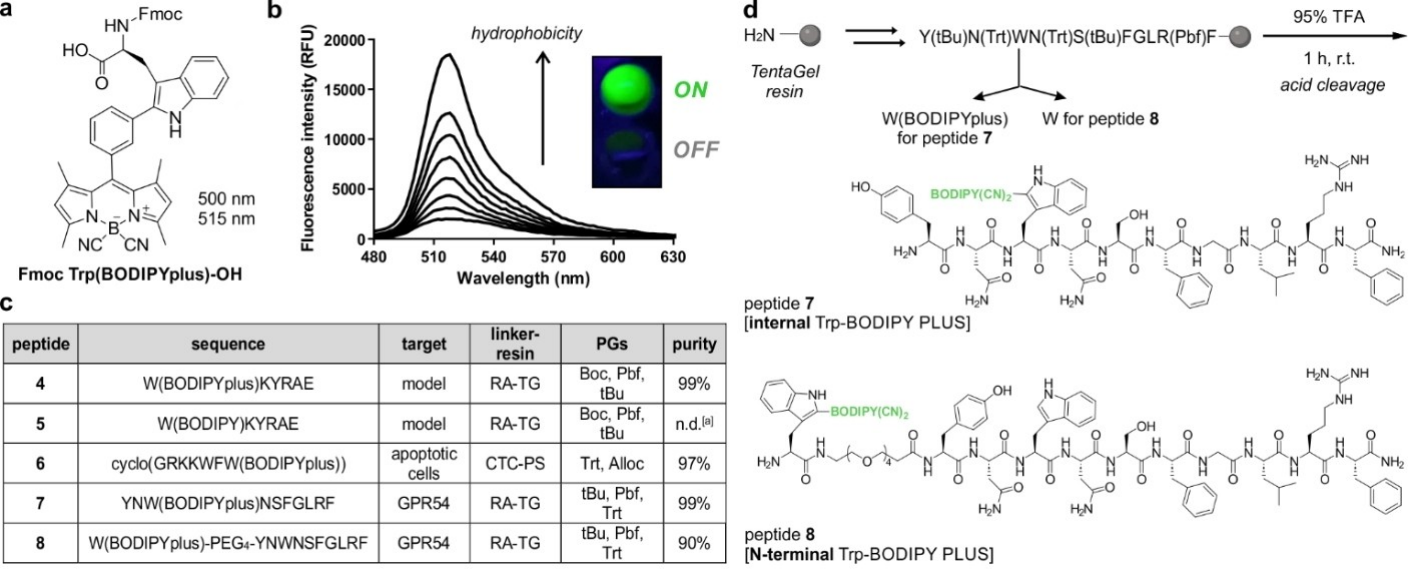
a) Chemical structure of the Fmoc‐protected Trp‐BODIPY PLUS amino acid for general peptide synthesis. b) Fluorescence emission spectra of Trp‐BODIPY PLUS (25 μM) in phosphate buffer saline (PBS) suspensions with increasing content of phosphatidylcholine liposomes. *λ*_exc_: 450 nm. Inset) UV‐irradiated pictograms of Trp‐BODIPY PLUS in hydrophilic (bottom) and hydrophobic (top) environments. c) Peptide sequences synthesized with Trp‐BODIPY and Trp‐BODIPY PLUS, their biological targets, solid supports, protected amino acids and purities after SPPS. RA: Rink Amide, TG: TentaGel, CTC: chlorotrityl, PS: polystyrene,^[a]^ not determined due to degradation of BODIPY. d) Synthetic scheme and structures of KP‐10 analogues with Trp‐BODIPY PLUS instead of the native Trp (peptide **7**) or at the N‐terminal end (peptide **8**).

Next, we studied the stability of Trp‐BODIPY PLUS in TFA and compared it to that of Trp‐BODIPY (Table 2). As expected, Trp‐BODIPY completely degraded after treatment with 95 % TFA for 5 min (<5 % stability); however, the cyano‐derivative Trp‐BODIPY PLUS was fully resistant to TFA even after 1 h (Table 2, Figure S1). This resistance can be due to the formation of hydrogen‐bonded complexes with TFA that stabilize the B−N bonds, as shown in other BODIPY dyes.^[14e]^ We also examined the integrity of Trp‐BODIPY PLUS under common SPPS conditions (Tables 2 and S2), which confirmed compatibility with multiple scavengers (e.g., triisopropylsilane (TIS), H_2_O, dithiothreitol (DTT)) and reagents (e.g., tetrabutylammonium fluoride (TBAF) to remove silyl‐based PGs, hydrazine for Dde group removal, Pd(PPh_3_)_4_ for Alloc removal and piperidine to remove Fmoc groups). Of note, Trp‐BODIPY PLUS degraded in the presence of iodine, as most BODIPY dyes.^[16]^ Finally, we evaluated the optical properties of Trp‐BODIPY PLUS. The replacement of fluorine atoms with cyano groups did not alter the absorbance and emission profiles (e.g., maxima wavelengths at 500 nm and 515 nm, respectively), and Trp‐BODIPY PLUS showed strong fluorogenic behavior (Figures 1 and S2–S5, Table S3). Previous TD‐DFT computational studies on phenyl‐BODIPY systems have shown that the accessibility to a conical intersection and subsequent nonradiative decay is controlled by a transition state on the first excited state, whose energy barrier defines the degree of fluorogenicity.[^10b^, ^17^] Trp‐BODIPY PLUS also displayed suitable solubility and photo‐ and chemical stability for long‐term storage (Figures S6–S8), thus representing a versatile building block for conventional SPPS of fluorogenic BODIPY peptides.

**Table 2:**
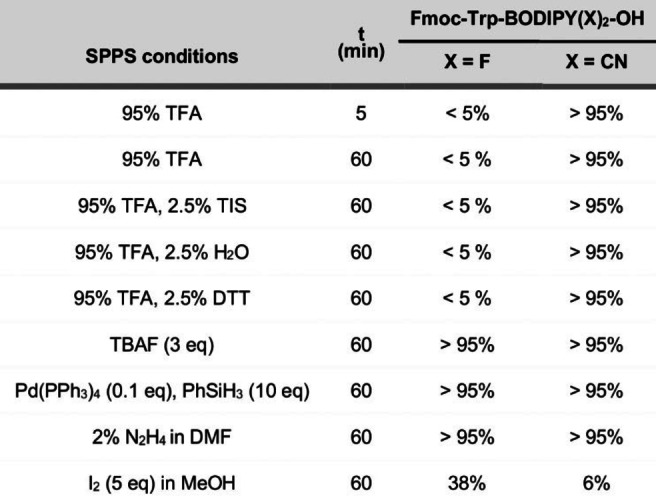
Stability analysis of Trp‐BODIPY FlAAs under SPPS conditions.^[a,b]^

[a] Reaction conditions: Trp‐BODIPY or Trp‐BODIPY PLUS (70 μM), DCM, r.t. [b] Purity determined by HPLC‐MS [%].

### Solid‐phase synthesis and evaluation of fluorogenic peptides using Trp‐BODIPY PLUS

To study the application of Trp‐BODIPY PLUS for SPPS of fluorogenic peptides, we first prepared analogues of the model linear sequence KYRAE, which contains a range of non‐polar, positively‐charged and negatively‐charged residues with different PGs (e.g., Boc, tBu, Pbf). We performed the synthesis using standard Fmoc/tBu SPPS conditions (e.g., OxymaPure and 1‐ cyano‐2‐ethoxy‐2‐oxoethylidenaminooxy)dimethylamino‐mor‐pholino‐carbenium hexafluorophosphate (COMU) for couplings, piperidine:DMF (2 : 8) for Fmoc removal) and introduced Trp‐BODIPY PLUS at the N‐terminal end. We performed the cleavage of the labeled peptide (i.e., W(BODIPYplus)KYRAE, peptide **4**, Figure 1) with 95 % TFA and compared the purity of the crude to that obtained when using the acid‐labile Trp‐BODIPY (i.e., W(BODIPY)KYRAE, peptide **5**, Figure 1). Notably, the HPLC‐MS analysis of both crude peptides after cleavage with 95 % TFA showed that the BODIPY core remained fully stable in peptide **4** whereas it was completely degraded in peptide **5** (Figure S9). Overall, we isolated the model peptide **4** in good yields >20 % and >95 % purity after HPLC purification (see Supporting Information for synthetic and characterization details).

Next, we assessed whether incorporation of Trp‐BODIPY PLUS enabled retention of the molecular recognition features of bioactive peptides. For this purpose, we derivatized the reported cyclic peptide Apo‐15 (cyclo(GRKKWFW(BODIPY))), which behaves as a fluorogenic probe for selective labeling of apoptotic cells.^[10e]^ Because Apo‐15 contains one Trp‐BODIPY residue within its sequence, we synthesized the corresponding analogue using Trp‐BODIPY PLUS as the FlAA. The resulting peptide **6** (Figure 1) was obtained with excellent yields and purities by SPPS using conventionally protected building blocks (e.g., K(Boc), R(Pbf)) and Rink Amide‐Tentagel resin as the solid support (Figure S10). We also examined the labeling properties of peptide **6** in co‐cultures of viable and apoptotic neutrophils using flow cytometry analysis. Notably, peptide **6** stained apoptotic cells ‐but not viable cells‐ within minutes and we confirmed its selectivity for apoptotic cells by co‐staining with the commercial marker AlexaFluor647 (AF647)‐Annexin V (Figures S11 and S12). Furthermore, like Apo‐15, the labeling of apoptotic cells with peptide **6** proved to be independent on the concentration of Ca^2+^ ions (Figure S13), which corroborates that the Trp‐BODIPY PLUS building block can be used to generate fluorogenic linear and cyclic peptides without impairing their bioactivity profiles.

With these results, we decided to employ Trp‐BODIPY PLUS to generate fluorogenic peptides for optical imaging of GPR54 in cells and tissues. GPR54 receptors bind truncated forms of the kisspeptin protein encoded by the KISS1 gene.^[18]^ The human full‐length kisspeptin protein is proteolytically cleaved into different peptides (e.g., KP‐54, KP‐14, KP‐13, KP‐10), which share a C‐terminal carboxamide.^[19]^ Among these, KP‐10 (YNWNSFGLRF) is the smallest active endogenous peptide against GPR54,^[20]^ thus we focused on the design of a new GPR54‐targeted fluorogenic probe (**7**, Figure 1) by replacing the native tryptophan residue in KP‐10 with the new Trp‐BODIPY PLUS. As optimized with the peptides **4** and **6**, we used standard SPPS to isolate peptide **7** in >95 % purity and suitable amounts for biological evaluation (see Supporting Information for synthetic and characterization details). Once the synthesis was completed, we determined the EC_50_ values of peptide **7** in human embryonic kidney (HEK293) cells stably expressing hGPR54. Specifically, we monitored the Ca^2+^ influx after incubation with the peptide and observed comparable agonist activity (EC_50_=2.5 nM) to that of the unlabeled peptide KP‐10 (EC_50_=0.22 nM, see Supporting Information for experimental details).

Notably, the replacement of the natural Trp residue by Trp‐BODIPY PLUS did not abolish the activity of the native peptide KP‐10, unlike previous studies with other fluorophores (e.g. Cy5.5) attached at the N‐terminal end.^[8a]^ Altogether, these results confirmed the applicability of Trp‐BODIPY PLUS as an acid‐resistant and fully SPPS‐compatible FlAA for the straightforward labeling of different peptide sequences with optimal fluorogenicity and minimal impact on the bioactivity profiles.

### Peptide 7 enables selective imaging of GPR54 in human cells

**Figure 2:**
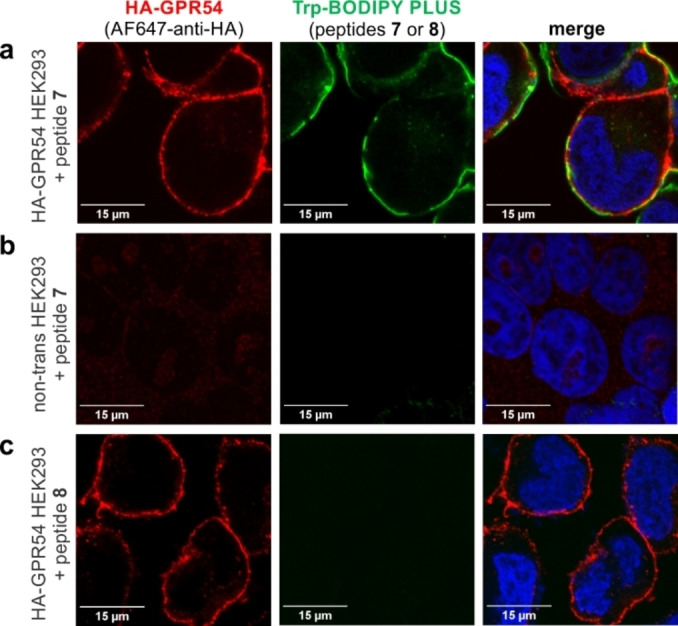
Representative fluorescence confocal microscopy images (from *n*=3) of HEK293 cells after incubation with peptides **7** and **8**. Transfected HEK293 cells expressing HA‐GPR54 were incubated with peptide **7** (green, 10 μM; panel a, middle) or peptide **8** (absence of green color, 10 μM; panel c, middle) followed by incubation with AF647‐anti‐HA antibody (red). Non‐transfected HEK293 cells that did not express HA‐GPR54 were incubated with peptide **7** (absence of green color, 10 μM; panel b, middle). DAPI was used as a nuclear counterstain (blue). Scale bars: 15 μm.

Having confirmed the agonist activity of peptide **7**, we next assessed its application for fluorescence imaging of HEK293 cells that stably expressed hGPR54 equipped with an additional N‐terminal hemagglutinin (HA) tag for dual color staining. First, we observed bright fluorescence labeling of transfected HEK293 cells after incubation with peptide **7** using fluorescence confocal microscopy (Figure 2a). Different concentrations of peptide **7** were tested (Figure S14), with optimal staining found at concentrations of 10 μM. In agreement with the expression of GPR54 on the surface of cells, the fluorescence signals of peptide **7** were mostly found on the membrane of HEK293 cells and colocalized with the far‐red fluorescence signals of an AF647‐anti‐HA antibody (Figure 2a, Figure S15). Furthermore, we confirmed the selectivity of peptide **7** for GPR54 receptors by performing experiments in control HEK293 cells that had not been transfected and did not express HA‐GPR54. In these cells, we observed complete lack of staining for both peptide **7** and anti‐HA under the same experimental conditions as above (Figure 2b, Figure S15).

Finally, we examined the relevance of the positioning of Trp‐BODIPY PLUS within the KP‐10 sequence. For this purpose, we synthesized an additional analogue of KP‐10 (peptide **8**, Figure 1) where Trp‐BODIPY PLUS was conjugated to the N‐terminal end via a short polyethylene glycol (PEG) spacer. We envisaged that peptide **8** would bind to GPR54 but emit weaker fluorescence signals because of the limited turn‐on effect of the Trp‐BODIPY PLUS when found away from the transmembrane region in a less hydrophobic environment. After the synthesis of peptide **8** (see Supporting Information for characterization data), we incubated transfected HEK293 cells with peptide **8**, and observed no green fluorescence signals despite confirming the expression of GPR54 by anti‐HA labeling (Figure 2c, Figure S15). These results confirmed that Trp‐BODIPY PLUS is an optimal reporter to prepare bioactive fluorogenic peptides because: 1) it can retain the binding ability of Trp‐containing peptides, and 2) its strong fluorogenic character can be used to directly monitor target binding. To the best of our knowledge, these are the first fluorogenic KP‐10 analogues for direct imaging of GPR54 receptors in human cells.

### Fluorescence imaging of GPR54 distribution in intact mouse pancreatic islets

GPR54 receptors are widely expressed in the hypothalamus as well as in peripheral tissues including liver and pancreas.^[21]^ In particular, kisspeptin is responsible for the regulation of insulin secretion from β cells in the pancreas and therefore it plays a key role in glucose metabolism.[^5^, ^22^] Insulin secretion primarily takes place in pancreatic islets, also known as islets of Langerhans.^[23]^ These islets comprise different types of endocrine cells, which include primarily insulin‐secreting β cells but also glucagon‐secreting α cells and somatostatin‐producing δ cells.^[24]^ The loss or dysfunction of β cells within pancreatic islets have been reported as contributing factors to both type 1 and 2 diabetes,^[25]^ highlighting the importance of optical tools to investigate pancreatic β cell function in real time.^[26]^ Furthermore, the downregulation of kisspeptin and GPR54 in the pancreas has been described in murine models of diabetes mellitus,^[21a]^ thus suggesting that this system is a potential therapeutic target to restore β cell function in people living with diabetes. In view of this, we decided to apply peptide **7** for imaging the distribution of GPR54 in mouse pancreatic islets.

**Figure 3:**
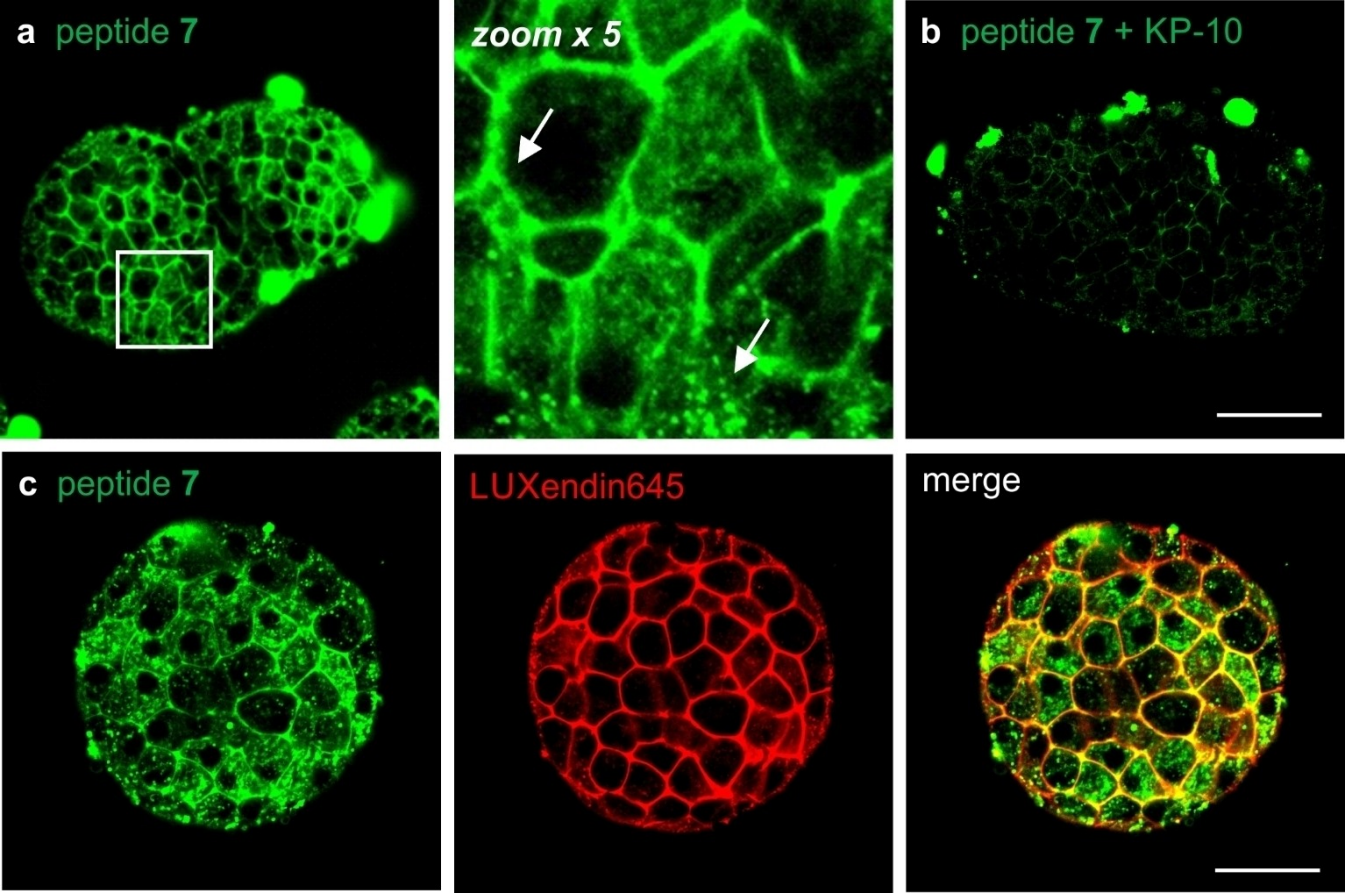
a) Representative fluorescence confocal microscopy images of live pancreatic islets (from *n*=3) upon incubation with peptide **7** (green, 10 μM) alone or b) peptide **7** (green, 10 μM) after 1 h pre‐incubation with the unlabeled analogue KP‐10 (50 μM). White arrows point surface and intracellular labeling. c) Representative fluorescence confocal microscopy images of live pancreatic islets (from *n*=3) after 1 h incubation with peptide **7** (green, 10 μM) and LUXendin645 (red, 100 nM). The membrane‐staining fraction of peptide **7** co‐localizes with the signals of LUXendin645. The agonistic behavior of peptide **7** is featured by the ligand‐mediated internalization of GPR54 receptors. Scale bars: a and *b*=53 μm, *c*=34 μm.

Pancreatic islets were harvested from wild‐type CD1 mice and cultured for 24–48 h at 37 °C. First, we incubated intact islets with different concentrations of the fluorogenic agonistic peptide **7** followed by fluorescence confocal microscopy. When islets were incubated for 1 h with peptide **7** (10 μM), we observed bright labeling of cell membranes as well as some bright intracellular puncta, highlighting the presence of some GPR54 receptors undergoing ligand‐mediated internalization (Figure 3a). To further corroborate this observation and the target specificity of peptide **7**, we performed competition experiments between the fluorogenic peptide and the unlabeled peptide sequence KP‐10. The competition between the two GPR54‐binding peptides led to a substantial reduction of the fluorescence signals in the cell membrane, suggesting that both peptide **7** and KP‐10 bind GPR54 to a similar extent in pancreatic islets (Figure 3b).

Finally, we decided to employ peptide **7** to investigate the abundance and nature of GPR54‐expressing cells within intact pancreatic islets. For these experiments, we performed two‐color imaging with peptide **7** and the commercially available LUXendin645, a red‐emitting fluorescent marker of glucagon‐like peptide 1 receptors (GLP1R) found at the surface of β cells and not α cells, the other major cell type found in pancreatic islets.^[27]^ The quantitative analysis of fluorescence microscopy images indicated that peptide **7** preferentially bound and internalized into β cells as most **7**‐labeled cells were also stained with LUXendin645 (peptide **7**+, LUX645+) (Figure S16). Furthermore, peptide **7** was not present in cells devoid of LUXendin645 (i.e., α cells that do not express GLP1R, Figure S16).^[28]^ These results suggest that the majority—but not all—β cells in pancreatic islets express GPR54 whereas these receptors are absent in other endocrine cells, such as α cells. To further evaluate the selectivity of peptide **7**, we also confirmed the lack of cross‐reactivity against several biologically relevant molecules, including different metabolites, H_2_O_2_ and glutathione (Figure S17). Altogether, peptide **7** represents one of the first fluorogenic probes to image and quantify the expression levels and localization of GPR54 in cells and whole tissues, opening new avenues to study the role of these receptors in multiple biological contexts.

## Conclusion

We have rationally designed Trp‐BODIPY PLUS as the first acid‐resistant BODIPY‐based amino acid for the construction of fluorogenic peptides under generic SPPS protocols. Trp‐BODIPY PLUS is stable in 95 % TFA and compatible with most SPPS reagents, and it exhibits a strong fluorogenic character that results in high signal‐to‐background ratios for both flow cytometry and confocal microscopy experiments. Importantly, Trp‐BODIPY PLUS can be incorporated into short linear and cyclic peptides without causing major alterations in their bioactivity profiles. We have demonstrated the application of Trp‐BODIPY PLUS to synthesize the new peptide **7** as the first fluorogenic analogue of the kisspeptin‐derived KP‐10 peptide for imaging of GPR54 receptors in live human and mouse cells, using simple and fast protocols. Peptide **7** selectively labels GPR54 in β cells within intact pancreatic islets, and its fluorescence signals can be used to quantitatively monitor their expression and internalization. Given the relevance of the GPR54/kisspeptin system in different pathologies (from cancer to type 2 diabetes), we envisage that peptide **7** will enable new real‐time mechanistic studies and accelerate the development of GPR54‐targeted therapeutics.

## Conflict of interest

The authors declare no conflict of interest.

1

## Supporting information

As a service to our authors and readers, this journal provides supporting information supplied by the authors. Such materials are peer reviewed and may be re‐organized for online delivery, but are not copy‐edited or typeset. Technical support issues arising from supporting information (other than missing files) should be addressed to the authors.

Supporting Information

## Data Availability

The data that support the findings of this study are available from the corresponding author upon reasonable request.
