# Supplementary material for: Acid‐Resistant BODIPY Amino Acids for Peptide‐Based Fluorescence Imaging of GPR54 Receptors in Pancreatic Islets
Source: Angew Chem Int Ed Engl. 2023 Apr 12;62(20):e202302688. doi: 10.1002/anie.202302688 (PMC10947197; doi:10.1002/anie.202302688)
Supplement: Supplementary file 1 — Supporting Information [file ANIE-62-0-s001.pdf]

Angewandte Chemie
Eine Zeitschrift der Gesellschaft Deutscher Chemiker

Supporting Information

Acid-Resistant BODIPY Amino Acids for Peptide-Based Fluorescence Imaging of GPR54 Receptors in Pancreatic Islets

L. Mendive-Tapia, L. Miret-Casals, N. D. Barth, J. Wang, A. de Bray, M. Beltramo, V. Robert, C. Ampe, D. J. Hodson, A. Madder, M. Vendrell**

Supplementary Information

Table of Contents

1. Supplementary Figures and Tables
2. Materials and Methods
3. Supplementary References

Supplementary Figures and Tables

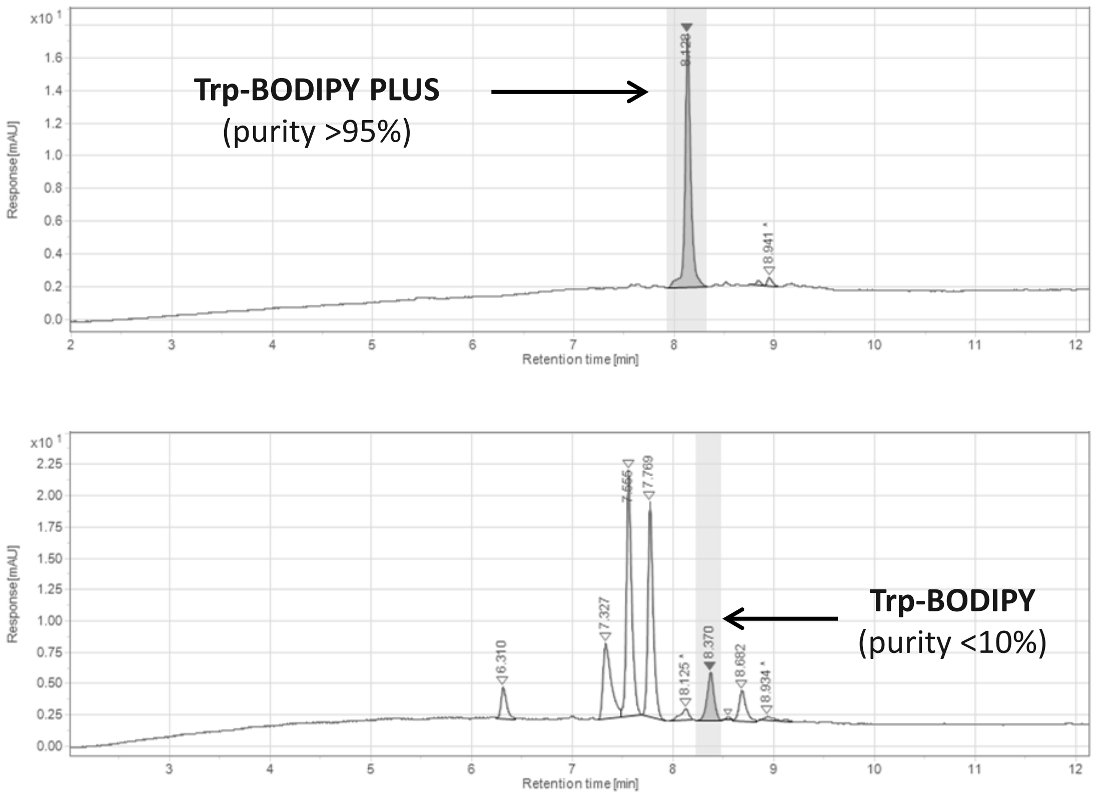

Supplementary Figure 1. HPLC traces of Trp-BODIPY and Trp-BODIPY PLUS after incubation with TFA:H_2O (95:5) for 5 min (Trp-BODIPY) and 60 min (Trp-BODIPY PLUS). UV detection: 500 nm.

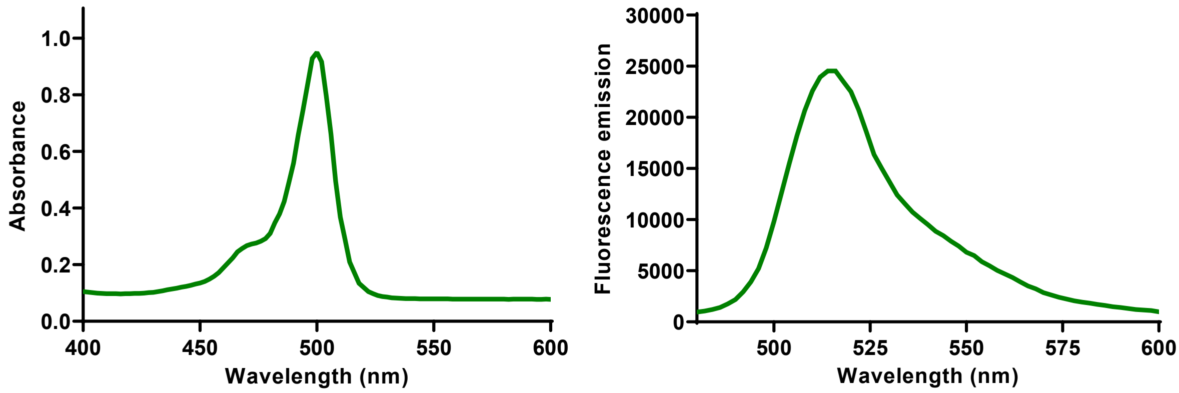

Supplementary Figure 2. Representative absorbance (left) and emission (right) spectra of Trp-BODIPY PLUS (25 µM) in EtOH. λ_{exc}: 450 nm (n=3).

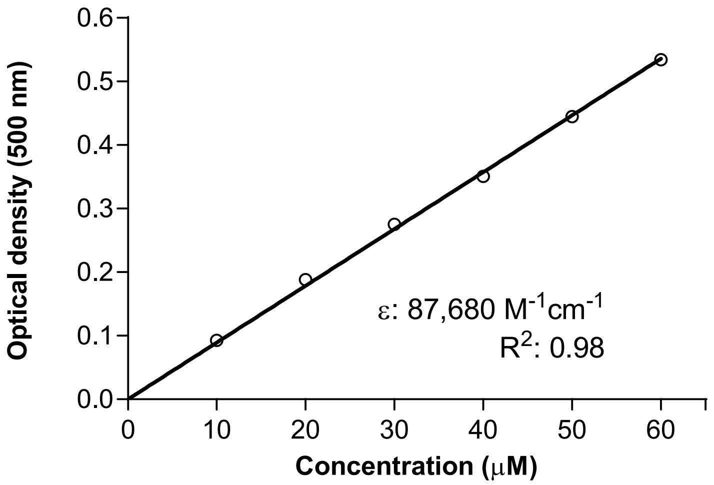

Supplementary Figure 3. Determination of the extinction coefficient of Trp-BODIPY PLUS in EtOH. Values presented as means±SEM (n=6).

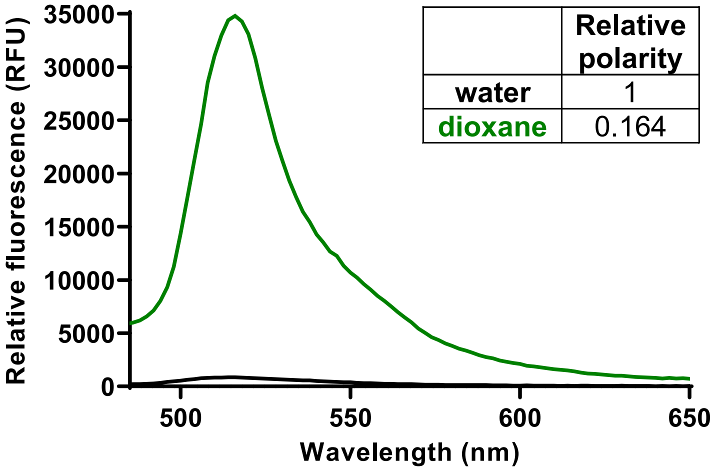

Supplementary Figure 4. Representative emission spectra of Trp-BODIPY PLUS (25 μM) in water (black) and dioxane (green). λ_{exc}: 450 nm (n=3).

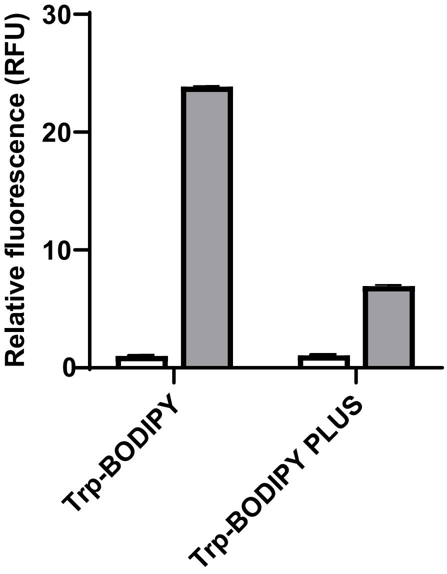

Supplementary Figure 5. Relative fluorescence intensity of Trp-BODIPY and Trp-BODIPY PLUS (10 μM) in phosphate buffer saline (PBS, white bars) and phosphatidylcholine liposome suspensions (grey bars). λ_{exc}: 450 nm; λ_{em}: 510 nm. Values presented as means±SEM (n=3).

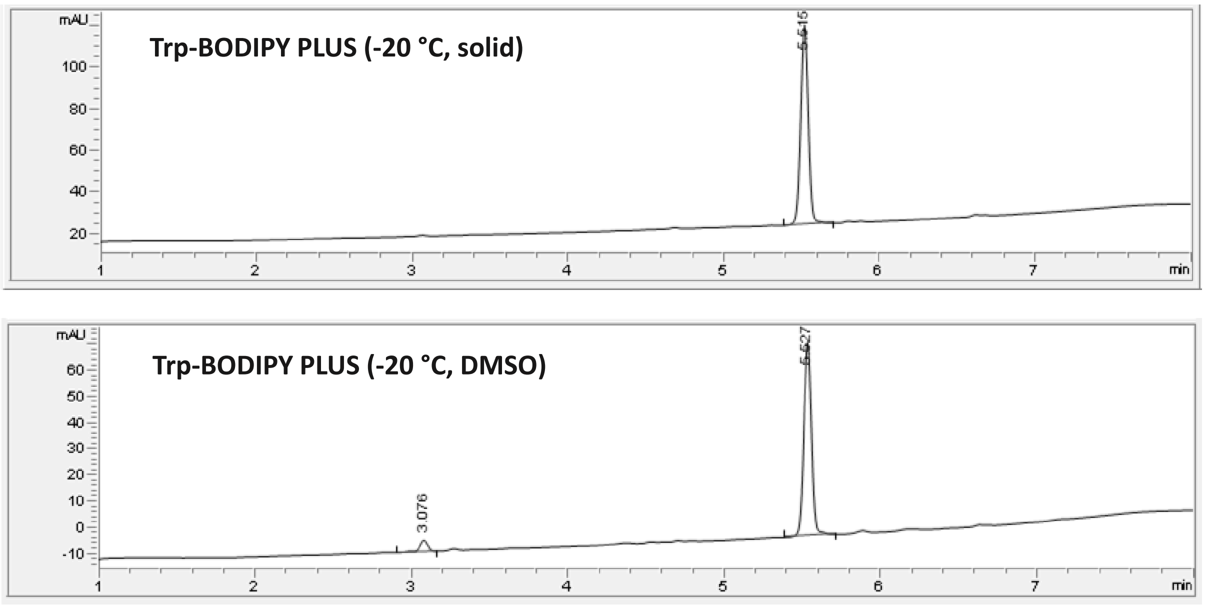

Supplementary Figure 6. HPLC traces of Trp-BODIPY PLUS after 5 months storage in the freezer as powder (top) and as 5 mM DMSO solutions (bottom).

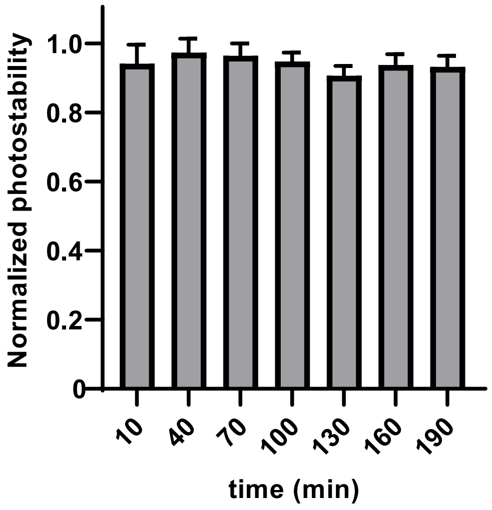

Supplementary Figure 7. Normalized fluorescence emission of Trp-BODIPY PLUS (50 μM, buffer) at 37°C under continuous light irradiation (Xe lamp, 20 W). λ_{exc}: 450 nm; λ_{em}: 510 nm. Values presented as means±SD (n=6).

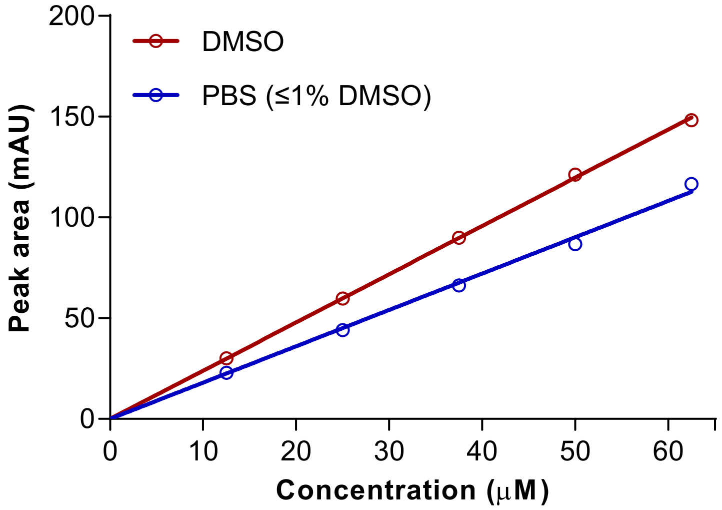

Supplementary Figure 8. Linear regressions of the peak absorbance area (mAU) of Trp-BODIPY PLUS in DMSO (red) and PBS (containing ≤1% DMSO, blue) at different concentrations of the amino acid. Values were determined by HPLC-MS using UV detection at 510 nm. R^2 values: 0.999 (red), 0.994 (blue).

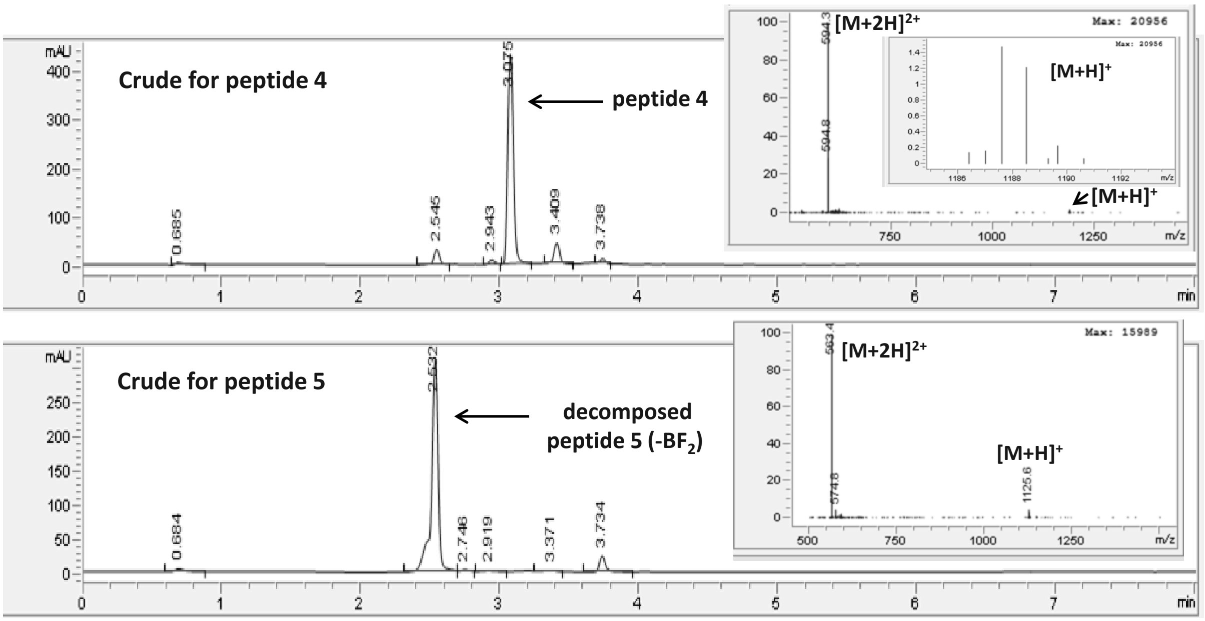

Supplementary Figure 9. HPLC-MS crude traces of peptides **4** and **5** after cleavage with TFA:TIS:H_2O (95:2.5:2.5) for 1 h. UV detection: 500 nm.

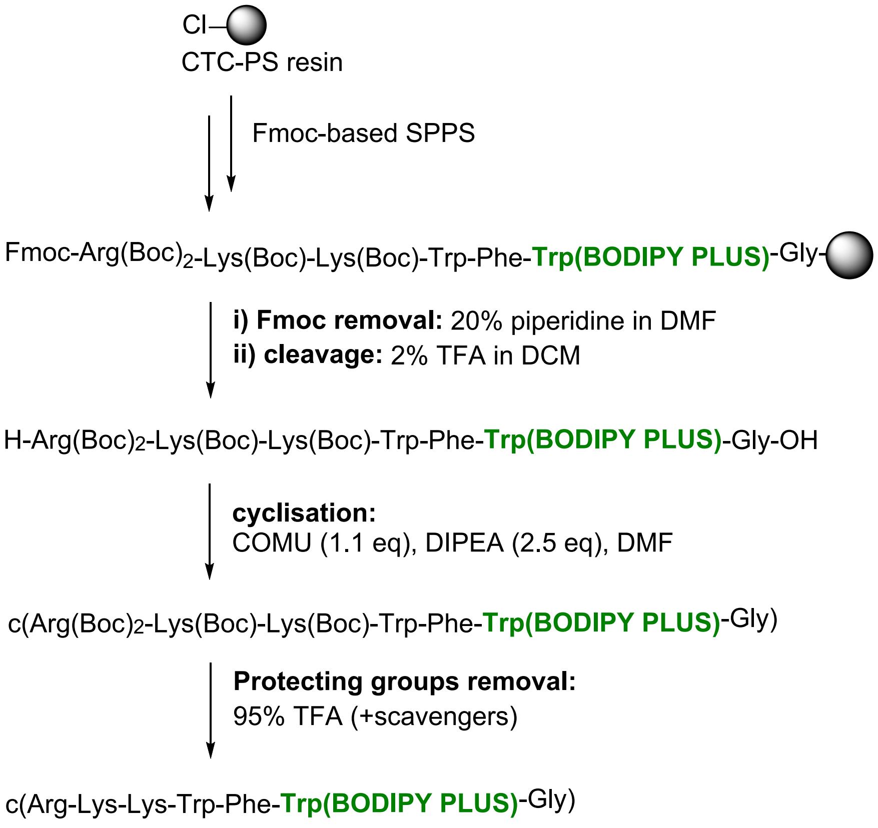

6

Supplementary Figure 10. Synthetic scheme for the preparation of peptide **6**.

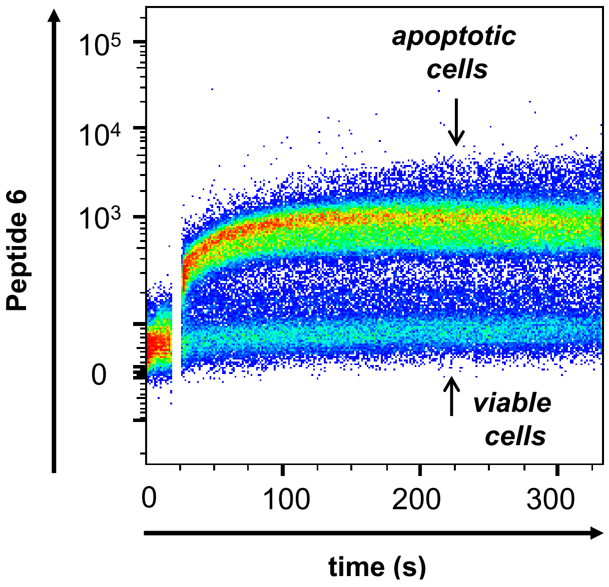

Supplementary Figure 11. Time-course fluorescence labeling of mixtures of apoptotic (top) and viable (bottom) neutrophils after incubation with peptide **6** (300 nM).

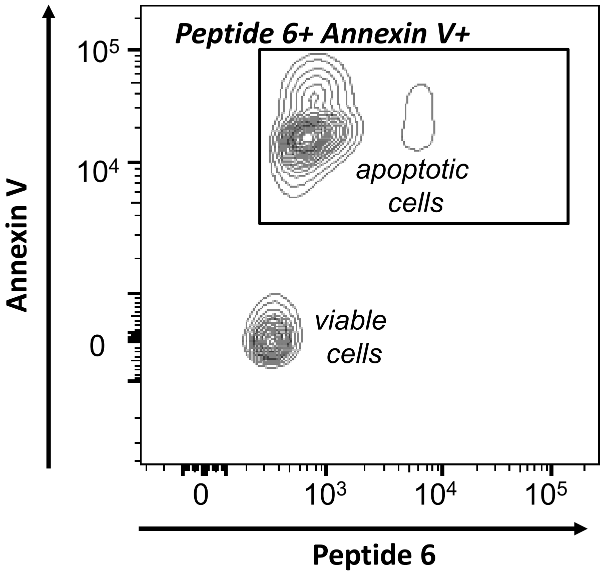

Supplementary Figure 12. Flow cytometric analysis of mixtures of viable cells and apoptotic human neutrophils upon incubation with peptide **6** (300 nM) and AF647-AnnexinV (25 nM).

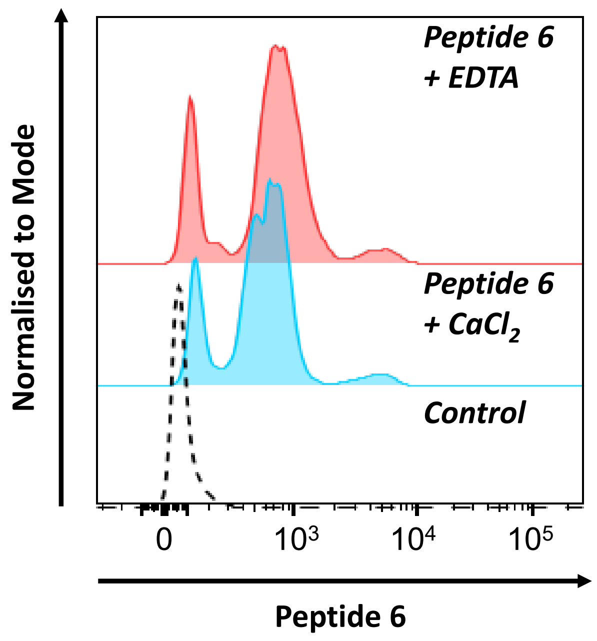

Supplementary Figure 13. Representative histograms showing labeling of apoptotic neutrophils after incubation with peptide **6** (300 nM) in buffer containing 2.5 mM EDTA (red, for Ca^{2+}-free conditions) and in buffer containing 2 mM $CaCl_2$ (blue).

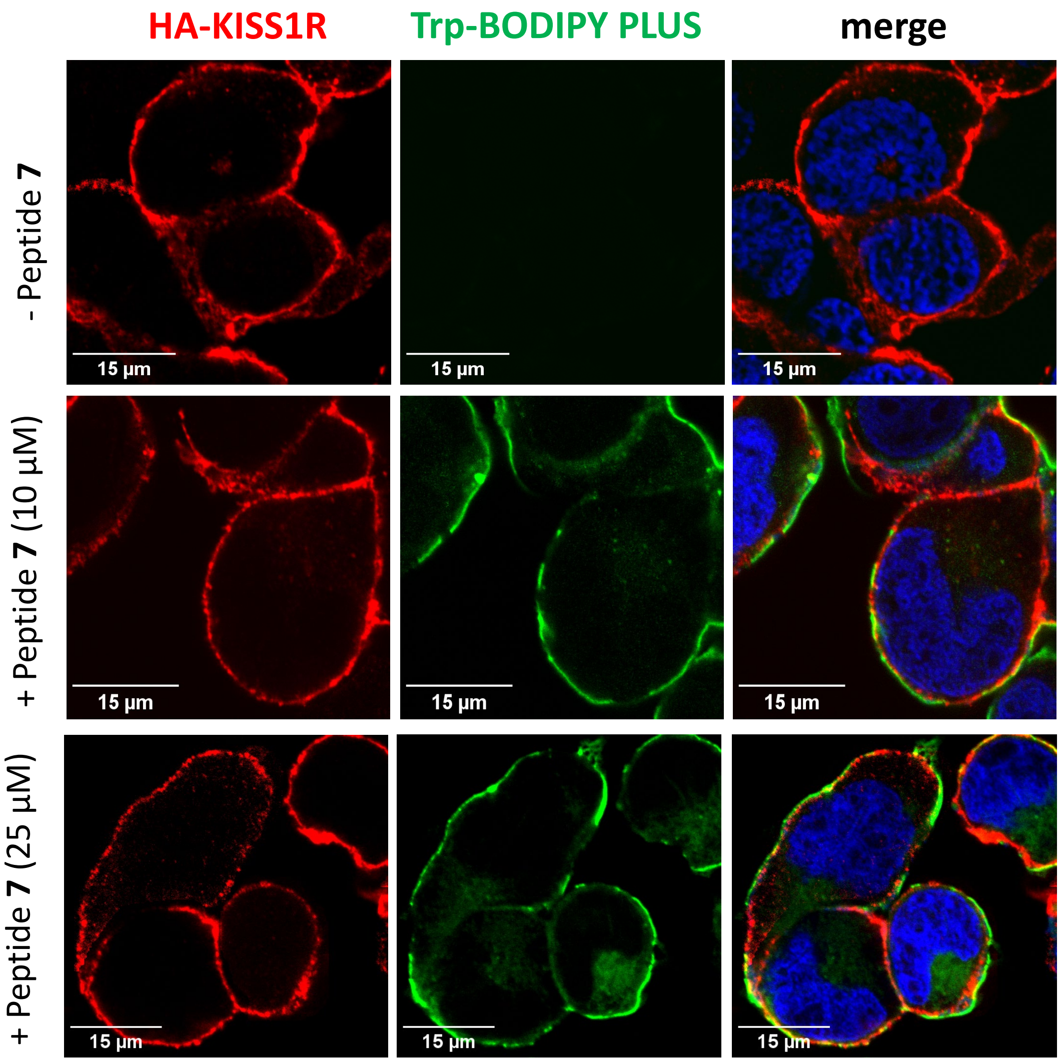

Supplementary Figure 14. Representative fluorescence confocal microscopy images (from 3 independent experiments) of HEK293 cells after incubation with peptide **7** at the indicated concentrations. Transfected HEK293 cells expressing HA-GPR54 were incubated with peptide **7** (green) for 30 min followed by incubation with AF647-anti-HA antibody (red). DAPI was used as a nuclear counterstain (blue). Scale bar: 15 µm.

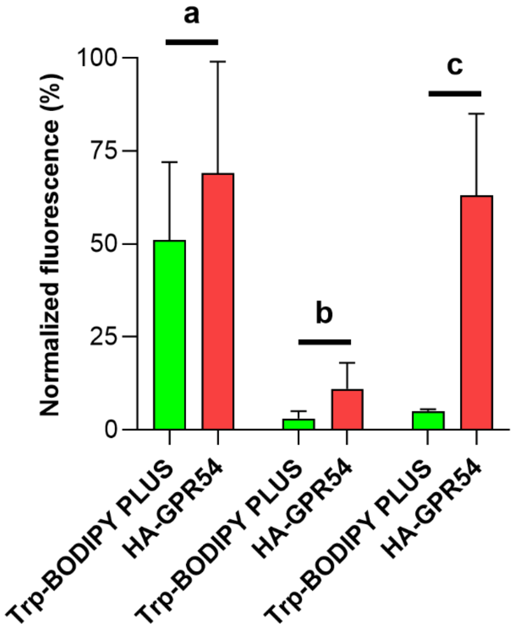

Supplementary Figure 15. Quantification of the fluorescence signals in HEK293 cells expressing or not HA-GPR54. a) Cells expressing HA-GPR54 after incubation with peptide **7** (green, 10 µM); b) non-transfected HEK293 cells incubated with peptide **7** (green, 10 µM); c) cells expressing HA-GPR54 after incubation with peptide **8** (green, 10 µM).

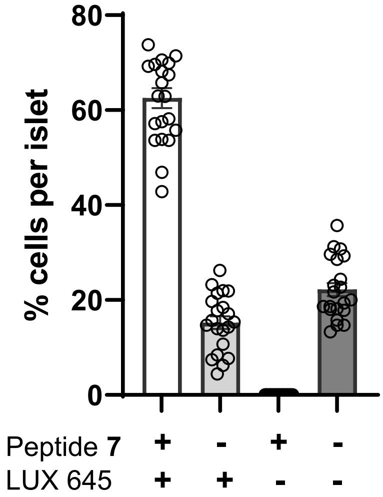

Supplementary Figure 16. Quantification of the percentage of cells per islet (n=21 islets from 3 independent animals) labelled with peptide **7** (10 µM) and/or LUXendin645 (LUX645, 100 nM).

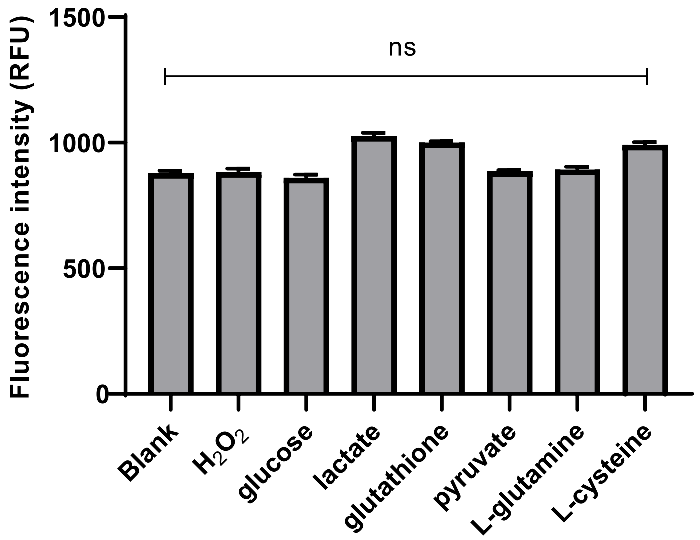

Supplementary Figure 17. Fluorescence intensity signals from peptide **7** upon incubation with H_2O_2 (10 mM), glucose (5 mM), lactate (2 mM), glutathione (1 mM), pyruvate (0.2 mM), L-glutamine and L-cysteine (2 mM). Data presented as means±SEM (n=3). P values for ns, p >0.05.

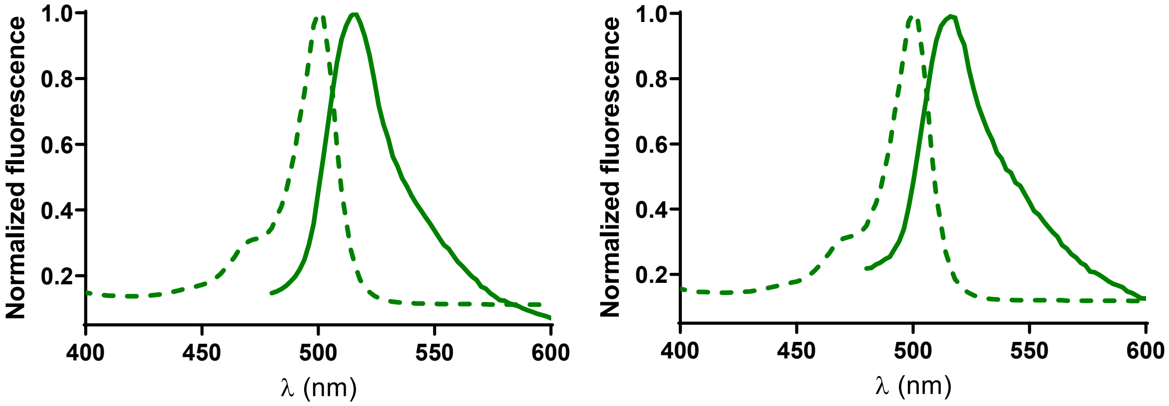

Supplementary Figure 18. Representative absorbance and emission spectra of peptides **7** (left, 25 µM) and **8** (right, 25 µM) in EtOH. λ_{exc}: 450 nm (n=3).

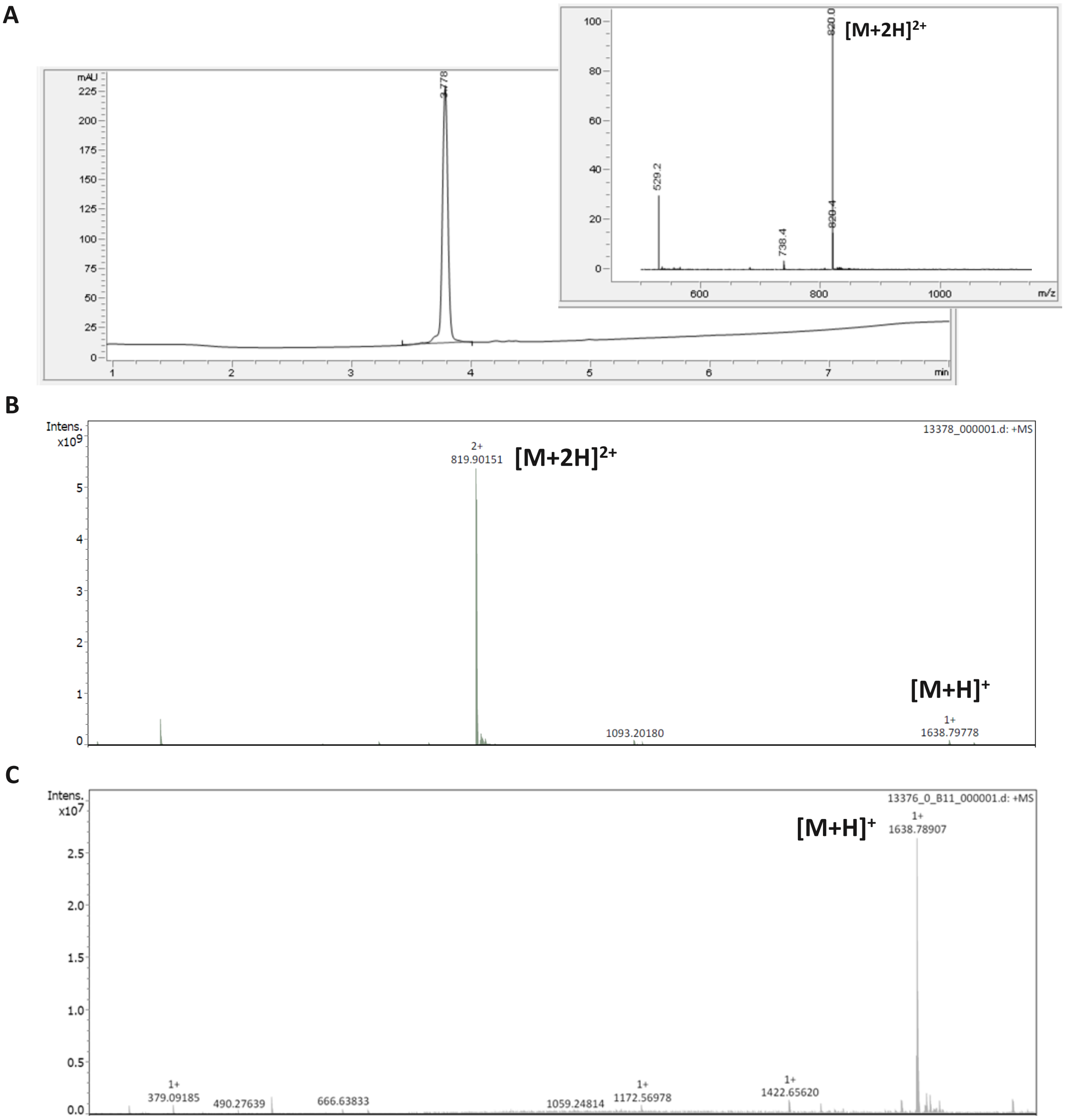

Supplementary Figure 19. A) HPLC-MS trace (UV detection: 254 nm), B) HRMS and C) MALDI spectrum of peptide **7**.

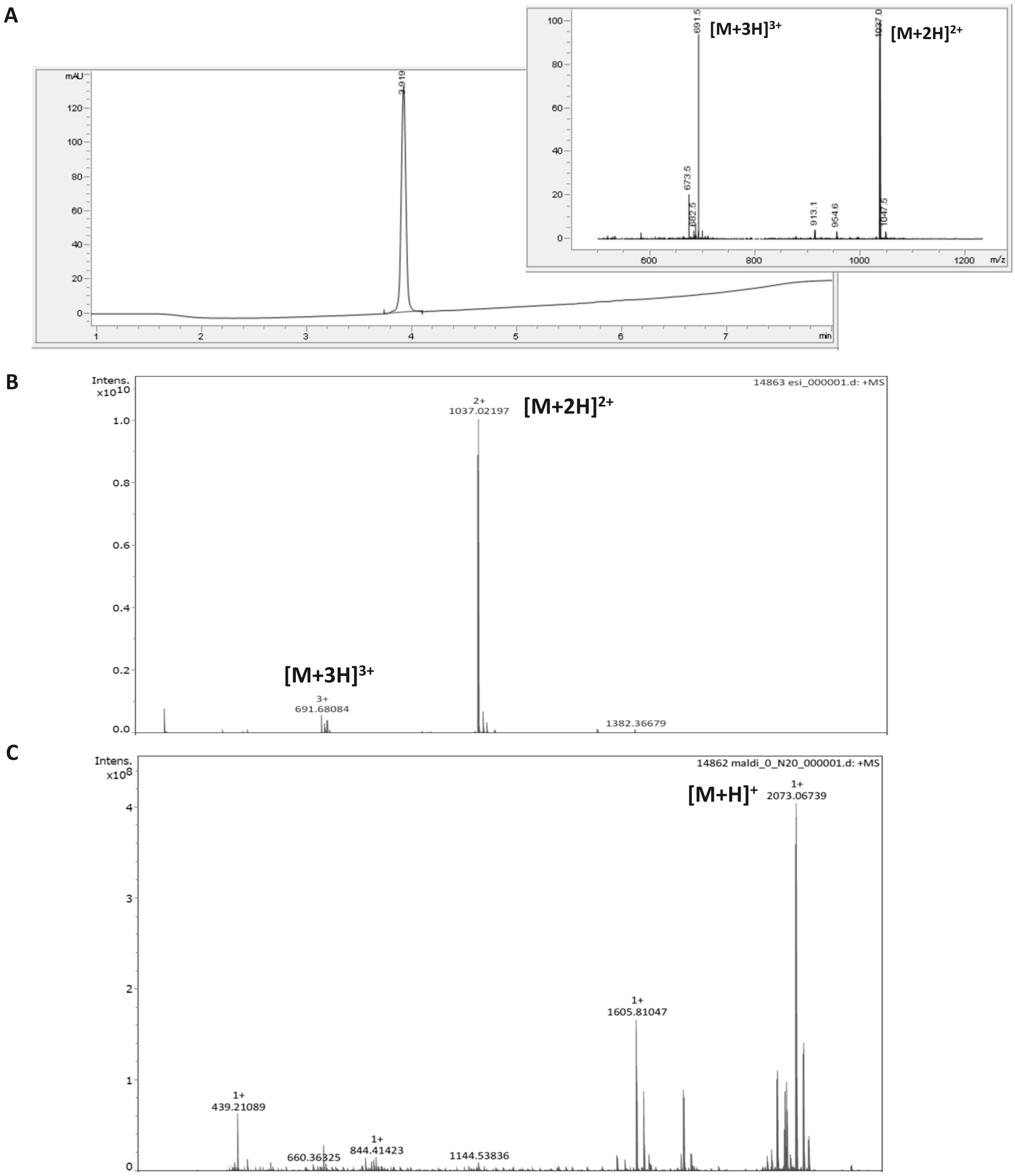

Supplementary Figure 20. A) HPLC-MS trace (UV detection: 254 nm), B) HRMS and C) MALDI spectrum of peptide **8**.

Supplementary Table 1. Evaluation of TMS-protected nucleophiles (TMS-Nu) to modify Trp-BODIPY.

TMS-Nu (eq)	Lewis acid (eq)	1 (%)	2 (%)	3 (%)	Other (%)
TMS-N_3 (20)	$SnCl_4$ (0.5)[a]	-	-	92.2	7.8
TMS-N_3 (15)	BCl_3 (2.0)[b]	79.7	-	1.3	19.0
TMS-OAc (10)	$SnCl_4$ (2.0)[c]	4.0	-	68.6	27.4
TMS-OAc (15)	BCl_3 (2.0)[b]	96.4	-	-	3.6
TMS-CF_3 (15)	BCl_3 (2.0)[b]	76.3	-	4.5	19.2

Reaction conditions: Trp-BODIPY (20 mM), DCM, 1 h, r.t. Conversion determined by HPLC-MS (%). [a]Trp-BODIPY (5 mM);[b] Et_3N (4 µL) was added to the reaction mixture;[c] 5 min, r.t.

Supplementary Table 2. Analysis of the chemical stability of Trp-BODIPY and Trp-BODIPY PLUS under conventional SPPS conditions. Amino acids were diluted with different reagent mixtures (70 μM, final volume 100 uL) and stirred at r.t. for 1 h (conditions A-B and E-H) or 5 min (conditions C and D). Mixtures were analyzed by HPLC-MS.

Entry	Conditions	Fmoc-Trp-BODIPY(X)$_2$-OH	
		X = F	X = CN
A	1% TFA, 2.5% DTT	> 95%	> 95%
B	5% TFA, 2.5% DTT	34%	> 95%
C	95% TFA, 2.5% DTT	7%	> 95%
D	20% piperidine:DMF	> 95%	> 93%
E	5% $NaHCO_3$ (aq)	> 95%	> 95%
F	5% Na_2CO_3 (aq)	> 95%	> 95%
G	1% DIPEA:DMF	> 95%	> 95%
H	0.1 M HOBt:TFE	> 95%	> 95%

Supplementary Table 3. Photophysical properties of Trp-BODIPY and Trp-BODIPY PLUS in EtOH.[1]

Compound	λ_{abs} (nm)	λ_{em} (nm)	ε (M^{-1} cm^{-1})	QY (%)
Trp-BODIPY PLUS	500	515	87,680	1.2
Trp-BODIPY	503	517	121,000	1.4

NMR spectra

^{1}H-NMR and ^{13}C-NMR spectra of Trp-BODIPY PLUS

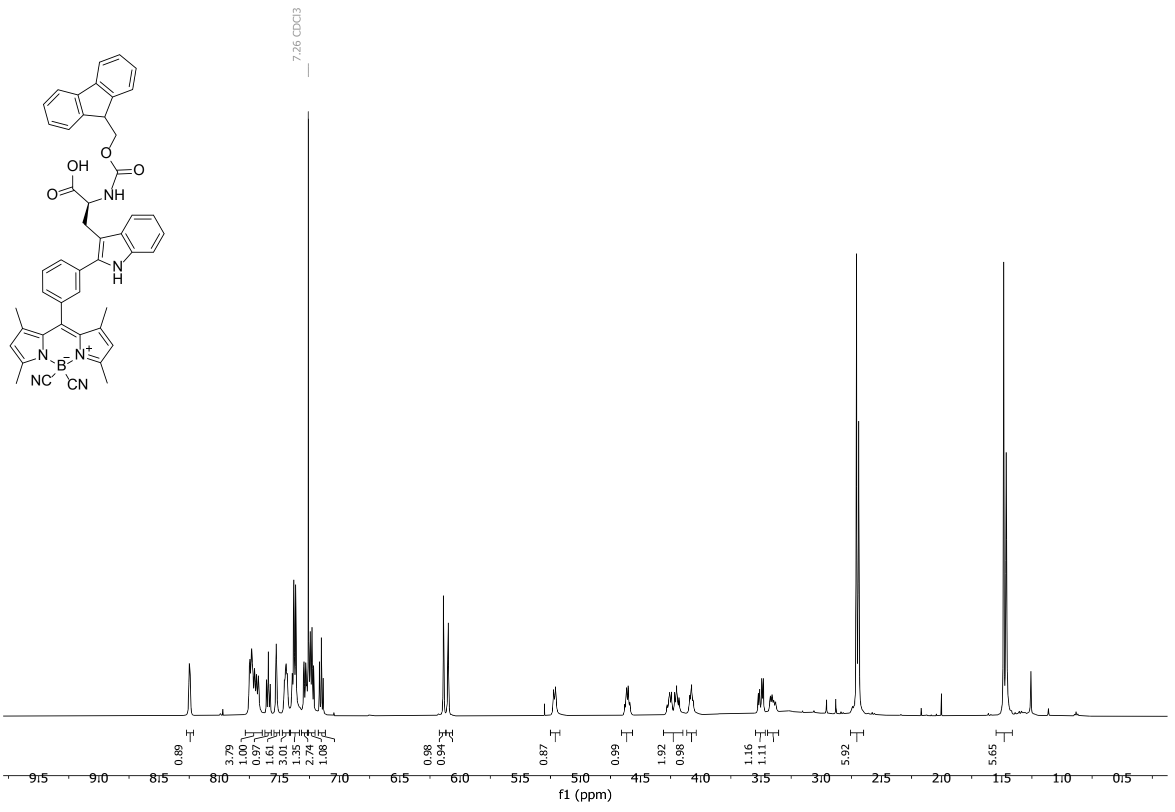

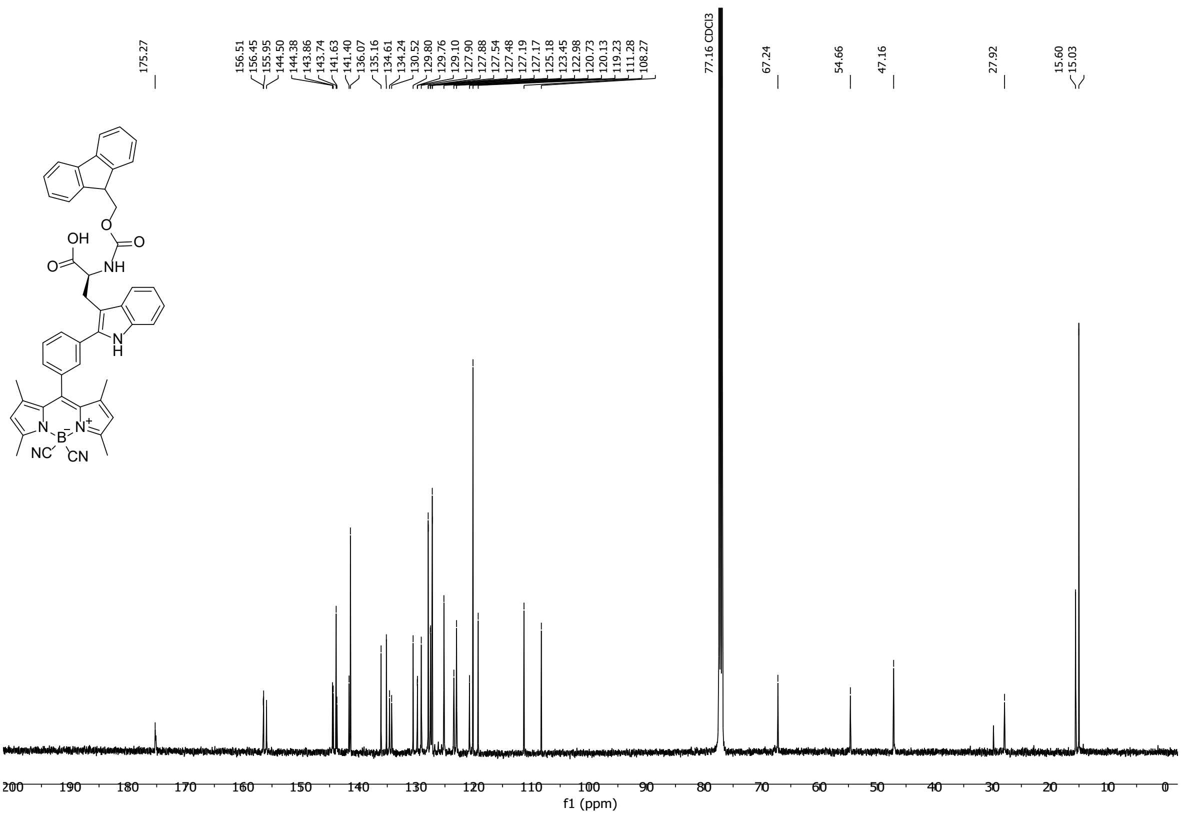

Materials and Methods

Chemicals were obtained from commercial sources and were used without further purification. Fmoc-Trp-BODIPY-OH was synthesized as described elsewhere.[2] Reactions were monitored by HPLC-MS analysis using a HPLC Agilent 1200 System comprising a Kinetex C_{18} column (5 µm, 100 Å, 150 x 4.6 mm) and a MS detector configured with an electrospray ionization source (6110 quadrupole LC/MS). A: H_2O (0.1% HCOOH) and B: CH_3CN (0.1% HCOOH) were used as eluents in a gradient from 0-100% B. Data acquisition was performed with MassLynx software. ^1H and ^{13}C spectra were recorded in a Bruker Avance 500 spectrometer (at 500 and 125 MHz, respectively). Data for ^1H NMR spectra are reported as chemical shift δ (ppm), multiplicity, coupling constant (Hz) and integration. Data for ^{13}C NMR spectra reported as chemical shifts relative to the solvent peak. HRMS (ESI positive) were obtained with a Bruker ESI Micro-TOF mass spectrometer. MALDI analysis was performed on a Bruker UltrafleXtreme MALDI TOF-TOF mass spectrometer. HPLC purifications were conducted in a semi-preparative Agilent HPLC consisting of a 1220 Infinity II autosampler and a 1260 Infinity II detector. Kinetex 150 × 21.2 mm (5 µm, 100Å) C18 column.

Experimental procedures and characterization data

Fmoc-Trp-BODIPY PLUS

Fmoc-Trp-BODIPY-OH (15.0 mg, 0.020 mmol) was weighted in a 10 mL round bottom flask and the system was purged with N_2. The amino acid was dissolved in 4.0 mL dry DCM followed by the addition of BF_3OEt_2 (24.7 µL, 0.20 mmol, 10 eq) and trimethylsilyl cyanide (52.3 µL, 0.40 mmol, 20 eq). The reaction was stirred at room temperature for 10 min (consumption of the starting material was monitored by HPLC-MS). The

reaction was then quenched with H_2O (7.5 mL), followed by DCM removal with N_2. The crude was extracted with AcOEt (10 mL) and the organic layer was washed with brine (x3). The combined organic layers were dried over anhydrous magnesium sulfate, filtrated, and concentrated under rotatory evaporator. Purification was conducted by semi-preparative HPLC using a 50-95% gradient over 17 min with detection at 254 and 500 nm. Pure fractions were collected and lyophilized to afford pure compound as a red solid (8.9 mg, 58%).

^{1}H NMR (500 MHz, $CDCl_3$): δ = 8.25 (s, 1H), 7.78 – 7.64 (m, 4H), 7.59 (t, *J* = 7.7 Hz, 1H), 7.53 (s, 1H), 7.45 (dd, *J* = 7.6, 4.6 Hz, 2H), 7.38 (m, 3H), 7.29 (d, *J* = 7.5 Hz, 1H), 7.23 (m, 3H), 7.15 (ddd, *J* = 8.0, 7.1, 1.0 Hz, 1H), 6.14 (s, 1H), 6.10 (s, 1H), 5.21 (d, *J* = 8.0 Hz, 1H), 4.66 – 4.57 (m, 1H), 4.31 – 4.15 (m, 2H), 4.08 (q, *J* = 5.9 Hz, 1H), 3.50 (dd, *J* = 14.6, 5.9 Hz, 1H), 3.40 (dd, *J* = 14.7, 7.7 Hz, 1H), 2.71 (s, 3H), 2.69 (s, 3H), 1.49 (s, 3H), 1.46 (s, 3H).

^{13}C NMR (126 MHz, $CDCl_3$): δ = 175.3, 156.5, 156.4, 156.0, 144.5, 144.4, 143.9, 143.7, 141.6, 141.4, 136.1, 135.2, 134.6, 134.2, 130.5, 129.8, 129.8, 129.1, 127.9, 127.9, 127.5, 127.5, 127.2, 127.2, 125.2, 123.4, 123.0, 120.7, 120.1, 119.2, 111.3, 108.3, 67.2, 54.7, 47.2, 27.9, 15.6, 15.0.

HRMS (ESI+) m/z calcd. for $C_{47}H_{39}BN_6O_4$ $[M+Na]^+$: 785.3018; found, 785.3010.

General procedures for SPPS[3]

Amino acids were obtained from Sigma-Merck, Cambridge Bioscience and Iris Biotech. DIC was obtained from Sigma-Aldrich. Rink Amide and chlorotrityl polystyrene resins were obtained from Rapp Polymer GmbH and Novabiochem, respectively. Unless otherwise indicated, peptides were manually synthesized in 2-10 mL polystyrene syringes fitted with porous polyethylene discs using common Fmoc-

SPPS protocols. Solvents, excess of reagents and soluble by-products were removed by suction. The Fmoc group was removed with piperidine/DMF (1:4) (1×1 min, 2×5 min), followed by DMF (×5), and DCM (×5) washes. All syntheses were carried out at r.t. Peptides bearing fluorescent moieties were always protected from light.

Resin loading for 2-chlorotrityl polystyrene resin. The first amino acid was loaded onto the resin using DIPEA (3.0 eq.) in DCM for 10 min followed by additional DIPEA (7.0 eq.) for extra 40 mins. MeOH (0.8 µL mg^{-1} resin) was added to cap remaining trityl groups. The resin was then filtered and washed using DCM (5×1 min) and DMF (5×1 min). The loading of the resin was determined by measuring the absorbance of piperidine-dibenzofulvene adduct at 290 nm.

Peptide elongation. After the Fmoc group was removed, the resin was washed with DMF (4×1 min), DCM (3×1 min), DMF (4×1 min). Coupling was carried out using Fmoc-AA-OH (4 eq.), COMU or DIPCDI coupling reagent (4 eq.), OxymaPure (4 eq.) in DMF for 1 h (+ DIPEA (8 eq.) for couplings with COMU). Coupling of Trp-BODIPY PLUS was carried out using Trp-BODIPY PLUS (1.2 eq.), COMU (1.2 eq.), OxymaPure (1.2 eq.) and DIPEA (2.4 eq.) in DMF. The resin was then washed with DMF (5×1 min), DCM (5×1 min) and filtered. The completion of the coupling step was confirmed using Kaiser Test. Before the next coupling cycle, Fmoc group is removed as described above.

Cleavage from resin for peptides **4-5** and **7-8**. The peptide was cleaved from the resin using 95% TFA, 2.5% TIS 2.5% H_2O for 1 h and washed with DCM (4×1 min). The combined filtrates were collected into a round bottom flask and concentrated under reduced pressure.

Cleavage from resin for peptide **6**. The peptide was cleaved from the resin using 1% TFA/DCM (5×1 min). The combined filtrates were collected into a round bottom flask containing DCM (20 mL) and concentrated under reduced pressure.

H-Trp(BODIPYplus)-Lys-Tyr-Arg-Ala-Glu-NH$_2$ (peptide 4)

The synthesis was performed on 70 mg of Rink Amide resin (0.18 mmol g^{-1}). Fmoc-Lys(Boc)-OH, Fmoc-Tyr(tBu)-OH, Fmoc-Arg(Pbf)-OH, Fmoc-Ala-OH and Fmoc-Glu(tBu)-OH were used as side-chain protected building blocks and their incorporation was carried on a Liberty Blue microwave peptide synthesizer (CEM). For that, DIC and OxymaPure reagents were used for each amide coupling and 20% piperidine in DMF was employed for the removal of Fmoc protecting groups. After cleavage as described above, the crude peptide was precipitated by adding cold Et_2O (dropwise) and the resulting precipitate was decanted and dried (×2). Purification was conducted by semi-Preparative HPLC using a 5-50% gradient over 17 min, with detection at 254 and 500 nm. Pure fractions were collected and lyophilized to afford peptide **4** as a red solid (3.4 mg, 23% yield).

HPLC-MS: t_R: 3.1 min (99% purity).

HRMS (ESI+) m/z calcd. for $C_{61}H_{75}BN_{16}O_9$ $[M-H]^-$: 1185.5923; found: 1185.5793. **MALDI** (m/z): $[M-H]^-$: 1185.5811.

cyclo(Gly-Arg-Lys-Lys-Trp-Phe-Trp(BODIPYplus)) (peptide 6)

The synthesis was performed on 40 mg of 2-chlorotrityl polystyrene resin (0.46 mmol g^{-1}). Fmoc-Gly-OH, Fmoc-Arg(Boc)$_2$-OH, Fmoc-Lys(Boc)-OH, Fmoc-Trp-OH and Fmoc-Phe-OH were used as building blocks. After cleavage as described above, the peptide crude was precipitated by adding cold Et_2O (dropwise) and the resulting solid

was decanted and dried to afford 24 mg of protected peptide (75% yield). 23 mg of cleaved peptide (1.0 eq) were then dissolved in DMF (0.026 M) with COMU (1.1 eq) and DIEA (2.5 eq) and the resulting mixture was stirred for 1 h at r.t. The peptide crude was purified by semi-Preparative HPLC using a 60-100% gradient over 25 min, with detection at 254 and 500 nm. The peptide was dissolved in TFA:DCM:DTT (95:2.5:2.5) for 1 h to remove the side-chain protecting groups, followed by precipitation in cold Et_2O (dropwise) and lyophilization to afford pure peptide **6** as an orange solid (2.2 mg, 13% yield from cyclization step).

HPLC-MS: t_R: 5.7 min (97% purity).

HRMS (ESI+) m/z calcd. for $C_{72}H_{85}BN_{18}O_7$ $[M+H]^+$: 1325.7014; found: 1325.6960.

MALDI (m/z): $[M+H]^+$: 1325.8600, $[M+Na]^+$: 1347.8444.

H-Tyr-Asn-Trp(BODIPYplus)-Asn-Ser-Phe-Gly-Leu-Arg-Phe-NH_2 (peptide 7)

The synthesis was performed on 56 mg of Rink Amide resin (0.18 mmol g^{-1}). Fmoc-Tyr(tBu)-OH, Fmoc-Asn(Trt)-OH, Fmoc-Ser(tBu)-OH, Fmoc-Phe-OH, Fmoc-Gly-OH, Fmoc-Leu-OH and Fmoc-Arg(Pbf)-OH were used as side-chain protected building blocks. Incorporation of the residues prior to Trp(BODIPY) PLUS were carried on a Liberty Blue microwave peptide synthesizer (CEM). For that, DIC and OxymaPure reagents were used for each amide coupling and 20% piperidine in DMF was employed for the removal of Fmoc protecting groups. After cleavage as described above, the crude peptide was precipitated by adding cold Et_2O (dropwise) and the resulting precipitate was decanted and dried (x2). Purification was conducted by semi-Preparative HPLC using a 5-60% gradient over 17 min, with detection at 254 and 500 nm. Pure fractions were collected and lyophilized to afford peptide **7** as an orange solid (3.3 mg, 20% yield).

HPLC-MS: t_R: 3.5 min (99% purity).

HRMS (ESI+) m/z calcd. for $C_{84}H_{100}BN_{21}O_{14}$ $[M+H]^+$: 1638.7924; found: 1638.7978.

MALDI (m/z): $[M+H]^+$: 1638.7891.

H-Trp(BODIPYplus)-PEG$_4$-Tyr-Asn-Trp-Asn-Ser-Phe-Gly-Leu-Arg-Phe-NH$_2$ (peptide 8)

The synthesis was performed on 100 mg of Rink Amide resin (0.69 mmol g^{-1}). Fmoc-Tyr(tBu)-OH, Fmoc-Asn(Trt)-OH, Fmoc-Trp(Boc)-OH, Fmoc-Ser(tBu)-OH, Fmoc-Phe-OH, Fmoc-Gly-OH, Fmoc-Leu-OH and Fmoc-Arg(Pbf)-OH were used as side-chain protected building blocks. The synthesis was performed on SYRO Multiple Peptide Synthesizer (Multisyntech) automated peptide synthesiser using the Fmoc strategy except for the incorporation of Fmoc-NH-PEG$_4$-OH linker and Trp-BODIPY PLUS. A mixture of 5 eq. amino acid in DMF (0.5 M), 5 eq. HBTU in DMF (0.5 M) and 10 eq. DIPEA in NMP (2 M) were added to the resin, with subsequent reaction for 40 min at r.t. Coupling of Fmoc-NH-PEG$_4$-OH (2 eq.) was carried out using HBTU (2 eq.) and DIPEA (4 eq.) in DMF for 1.5 h. Coupling of Trp-BODIPY PLUS (1.5 eq.) was carried with HBTU (1.5 eq.) and DIPEA (4 eq.) in DMF for 1.5 h. After cleavage as described above, the crude peptide was precipitated by adding cold Et_2O (dropwise) and the resulting precipitate was decanted and dried (x3). Purification was conducted by semi-Preparative HPLC using a 0-100% gradient over 25 min, with detection at 254 nm. Pure fractions were collected and lyophilized to afford peptide **8** as a red solid (1.0 mg, 2% yield).

HPLC-MS: t_R: 4.7 min (90% purity).

MS (ESI+) m/z calcd. for $C_{114}H_{199}BN_{39}O_{29}$ $[M+H]^+$: 2071.0, found: 2071.3.

Spectroscopy measurements. Optical properties were measured in a Synergy H1 BioTek spectrophotometer at the indicated concentrations on 96 or 384-well plates. Absorbance and emission spectra were determined in the range of 400–700 nm (every 2 nm). For liposome experiments, phosphatidylcholine (PC)-based liposomes were purchased from Liposoma, diluted in PBS, and transferred into a black flat-bottom 96-well plate for optical measurements. For extinction coefficient measurements, the absorbance of each sample at the maximum excitation wavelength was recorded and the extinction coefficient was determined by fitting the data to Beer's law.

Cell experiments. Work with human peripheral blood leukocytes complied with all relevant ethical regulations and informed consent was obtained. The study protocol was approved by the Medical Regional Ethics Committee (21-EMREC-041) at the University of Edinburgh. Human blood leukocytes were isolated from healthy volunteers. Briefly, whole blood was anti-coagulated with sodium citrate [0.4% (w/v) final concentration] and centrifuged at 350 *g* for 20 min. Platelet-rich plasma was removed and autologous serum was generated by recalcification with $CaCl_2$ (20 mM final concentration). Erythrocytes were separated from leukocytes by sedimentation with Dextran T500 (0.6%) for 30 min and leukocyte populations were further fractionated using isotonic Percoll (GE Healthcare) 49.5%/63%/72.9% discontinuous gradients. Polymorphonuclear cells (>95% neutrophils) were harvested from the 63%/72.9% interface. Neutrophil apoptosis was induced by in vitro culture for 18 h at 37 °C and 5% CO_2 in IMDM supplemented with 5% autologous serum.

HEK293 cells. Immortalized human embryonic kidney cells HEK293 were grown in DMEM without pyruvate with 4.5 g L^{-1} of D-glucose, FBS, and penicillin/streptomycin

at 37 °C in a humidified atmosphere of 5% CO_2. HEK293 cells stably expressing hGPR54 receptor with an HA tag at the N-terminus were used in this study. Wild-type human GPR54 was cloned into the pcDNA3.1 expression vector in fusion at its 5' end with a HA tag. HA amino acid sequence: YPYDVPDYA (Tyr-Pro-Tyr-Asp-Val-Pro-Asp-Tyr-Ala). The resulting construct was transfected in HEK293 cell line and selected for stable expression using Geneticin. HEK293 transfected cells were grown in DMEM without pyruvate with 4.5 g L^{-1} of D-glucose, FBS, Geneticin® (200 μg μL^{-1} final concentration), and penicillin/streptomycin at 37 °C in a humidified atmosphere of 5% CO_2.

Flow cytometry. Peptide **6** was reconstituted at 5 mM in DMSO and used at 300 nM in HEPES-NaCl buffer containing 2 mM $CaCl_2$, with or without 2.5 mM EDTA, and were incubated with the cells for 10 min at r.t. before flow cytometry analysis, unless otherwise stated. AF647-Annexin V was used at the indicated concentration and added to cells 10 min prior to analysis. Fluorescence emission was measured on a 5 L LSR flow cytometer (BD). Excitation sources/emission filters used: peptide **6** (488 nm/525 nm); AF647-Annexin V (641 nm/670 nm). Data was analyzed with FlowJo software.

Calcium mobilization assays. Experiments were performed using a HEK293 cell line stably expressing human GPR54. Cells were grown in DMEM (with Glutamax, high glucose and without pyruvate) 10% fetal calf serum, 1% penicillin, 1% streptomycin, 200 μg mL^{-1} geneticin and HEPES (25 mM). The dynamics of intracellular Ca^{2+} mobilization induced by treating the cells with KP-10 and peptide **7** was monitored using the Fluo4 NW Ca^{2+} assay kit. Cells were plated 48 h before the experiment into

96-well black plates at a concentration of 50,000 cells per well. The day of the experiment, peptides were diluted from 1 M in DMSO in LoBind Tubes or in non-binding plates to the final desired concentration. The media was removed, and cells were rinsed with PBS, and incubated with the dye for 30 min at 37 °C and 30 min at r.t. Fluorescence was measured with a plate reader and data points were fitted to sigmoidal curves using GraphPad Prism 9.

Confocal microscopy of HEK293 cells. HEK293 cells stably expressing or not hGPR54 (25,000 cells per well) were seeded in 8-well ibidi chambers two days before performing the confocal microscopy experiments. The cells were treated with peptides **7** and **8** at 10, 25, and 50 µM for 30 minutes at 37 °C. Then, the cells were washed with PBS (no Ca^{2+} and Mg^{2+}) and 4% paraformaldehyde in PBS (no Ca^{2+} and Mg^{2+}) was added for 15 minutes to fix the cells. PBS with no Ca^{2+} and Mg^{2+} is always used for the rest of the protocol. The cells were washed with PBS (×3) and blocked with 1% BSA (with 0.1% Tx100 for permeabilization of the cell membrane) in PBS for 1 h at r.t. Then, the cells were washed (×3) with PBS and treated with the primary antibody (Anti-HA produced in rabbit) in 1% BSA in PBS (1:1000 dilution) overnight at 4 °C. Cells were washed with PBS (×3) and incubated with the secondary antibody (AF647 anti-rabbit IgG, 1:10000 dilution) in 1% BSA in PBS for 1 h at r.t. Finally, cells were washed with PBS and treated with 4',6-diamidino-2-phenylindole dihydrochloride (DAPI, 1:1000 in PBS) for 2 min. Confocal microscopy was performed on an Olympus FV1000 confocal laser scanning unit, combined with the Olympus IX81 inverted microscope. AF647 (red channel to detect the GPR54) was excited by the 633 nm laser and detected through a 640-680 nm emission filter. Trp-BODIPY PLUS was excited by the 488 nm and detected through a 500-530 nm emission filter. DAPI was excited by the

405 nm laser and detected through a 380-420 nm emission filter. Images were then processed with ImageJ software.

Animal studies. Animal studies were regulated by the Animals (Scientific Procedures) Act 1986 of the UK (PP1778740). Approval was granted by the University of Birmingham's Animal Welfare and Ethical Review Body (AWERB). Female 8-12 weeks-old CD1 or C57BL/6 mice were used as wild-type tissue donors. Mice were socially housed in SPF conditions under 12 h light-dark cycles with *ad libitum* access to food and water, relative humidity 55±10% and temperature 21±2 °C.

Primary islet isolation. Animals were euthanised in accordance with a schedule 1 procedure and bile ducts injected with Serva NB8 1 mg mL^{-1} collagenase. The pancreas was dissected, islets isolated using a histopaque gradient and cultured at 37°C, 5% CO_2 in RPMI supplemented with 10% FBS, 1% penicillin/streptomycin and 1% glutamine.

Peptide 7 and KP-10 competition assays. 10-12 medium to large WT islets were picked 48-72 h after isolation into a plastic, round-bottomed 96-well plate into wells containing complete RPMI with 50 µM KP-10 and incubated for 1 h, 37°C, 5% CO_2. Islets were then transferred to a well with 100 µL complete RPMI with 10 µM peptide **7** and incubated for 1 h at 37°C, 5% CO_2. For control, non-competition assay wells, islets were incubated for 1 h in 100 µL complete RPMI with: a) 10 µM peptide **7**, b) 10 µM KP-10, c) no probes. Islets were washed with complete RPMI prior to transfer to a glass, flat-bottomed 96-well imaging plate for imaging in complete RPMI.

Islet labeling with LUXendins. LUXendins are GLP1R markers[4] used to identify GLP1R+ cells. 12-15 medium to large WT islets were picked 24-72 h after isolation into a plastic, round-bottomed 96-well plate into wells containing complete RPMI with: a) 100 nM LUXendin645 and 10 µM peptide **7**, b) 100 nM LUXendin645 and 10 µM KP-10, c) 100 nM LUXendin645, d) no probes. Islets were incubated in these solutions for 1 h at 37°C, 5% CO_2 and washed with complete RPMI prior to transfer to a glass, flat-bottomed 96-well imaging plate for imaging in complete RPMI.

Live imaging of islets. Live imaging of cells and islets was performed with Zeiss LSM780/LSM880 meta-confocal microscopes equipped with sensitive GaAsP spectral detectors and 40x and 63x/1.2 W Korr FCS M27 objectives. Excitation was delivered at 488 nm (peptide **7** and KP-10) and 633 nm (LUXendin645) and signals were detected at 496-604 nm (peptide **7** and KP-10), 639-693 nm (LUXendin645). Representative images had linear adjustments applied to brightness and contrast and to enable cross-comparison, intensity values were maintained between samples. Fluorescence intensity was measured using a ROI and mean intensity in ImageJ.

Supplementary References

[1] L. Mendive-Tapia, C. Zhao, A. R. Akram, S. Preciado, F. Albericio, M. Lee, A. Serrels, N. Kielland, N. D. Read, R. Lavilla, M. Vendrell, *Nat. Commun.* **2016**, *7*, 10940.

[2] L. Mendive-Tapia, R. Subiros-Funosas, C. Zhao, F. Albericio, N. D. Read, R. Lavilla, M. Vendrell, *Nat. Protoc.* **2017**, *12*, 1588-1619.

[3] W. C. Chan, P. D. White, *Fmoc solid phase peptide synthesis: a practical approach*, Oxford University Press, Oxford, UK, **2000**.

[4] J. Ast, A. Arvaniti, N. H. F. Fine, D. Nasteska, F. B. Ashford, Z. Stamataki, Z. Koszegi, A. Bacon, S. Trapp, B. J. Jones, B. Hastoy, A. Tomas, C. A. Reissaus, A. K. Linnemann, E. D'Este, D. Calebiro, K. Johnsson, T. Podewin, J. Broichhagen, D. J. Hodson, *Nat. Commun.* **2020**, *11*, 467.
